# Metabolic Symbiosis Enables Adaptive Resistance to Anti-angiogenic Therapy that Is Dependent on mTOR Signaling

**DOI:** 10.1016/j.celrep.2016.04.029

**Published:** 2016-04-28

**Authors:** Elizabeth Allen, Pascal Miéville, Carmen M. Warren, Sadegh Saghafinia, Leanne Li, Mei-Wen Peng, Douglas Hanahan

**Affiliations:** 1 The Swiss Institute for Experimental Cancer Research (ISREC), EPFL SV ISREC, Station 19, 1015 Lausanne, Switzerland; 2 The Institute of Chemical Sciences and Engineering (ISIC-SB-EPFL), Ecole Polytechnique Fédérale de Lausanne, EPFL SB ISIC-Direction, CH A3 398 Station 6, 1015 Lausanne, Switzerland; 3 Department of Molecular, Cell, and Developmental Biology, University of California, Los Angeles, CA 90095, USA; 4 The Swiss Cancer Center Lausanne (SCCL), Swiss Federal Institute of Technology Lausanne (EPFL), 1015 Lausanne, Switzerland

## Abstract

Therapeutic targeting of tumor angiogenesis with VEGF inhibitors results in demonstrable, but transitory efficacy in certain human tumors and mouse models of cancer, limited by unconventional forms of adaptive/evasive resistance. In one such mouse model, potent angiogenesis inhibitors elicit compartmental reorganization of cancer cells around remaining blood vessels. The glucose and lactate transporters GLUT1 and MCT4 are induced in distal hypoxic cells in a HIF1α-dependent fashion, indicative of glycolysis. Tumor cells proximal to blood vessels instead express the lactate transporter MCT1, and p-S6, the latter reflecting mTOR signaling. Normoxic cancer cells import and metabolize lactate, resulting in upregulation of mTOR signaling via glutamine metabolism enhanced by lactate catabolism. Thus, metabolic symbiosis is established in the face of angiogenesis inhibition, whereby hypoxic cancer cells import glucose and export lactate, while normoxic cells import and catabolize lactate. mTOR signaling inhibition disrupts this metabolic symbiosis, associated with upregulation of the glucose transporter GLUT2.

## Highlights

• Angiogenesis inhibitors causing acute hypoxia elicit metabolic compartmentalization
• Hypoxic cancer cells import and metabolize glucose, secreting lactate
• Normoxic vessel-proximal cancer cells import and metabolize lactate, involving mTOR
• Co-inhibiting mTOR disrupts the metabolic symbiosis

## Graphical Abstract

**Figure:**
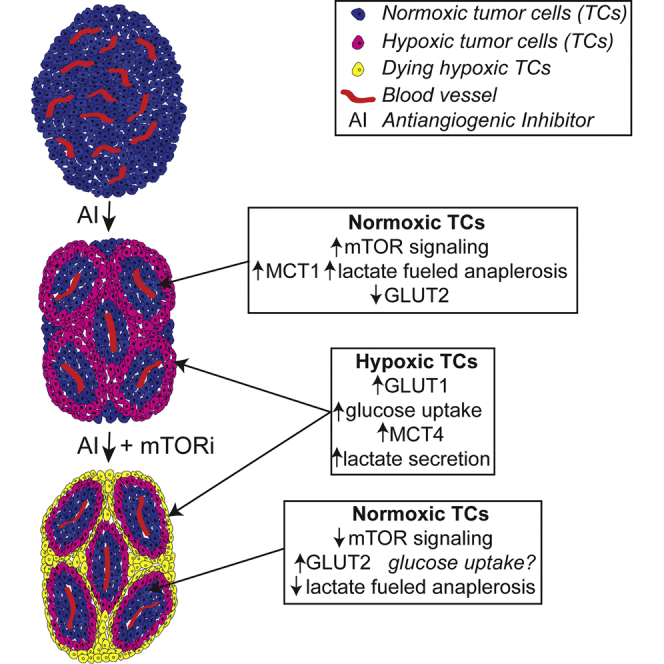

## Teaser

Allen et al. find that inhibitors of tumor angiogenesis that cause acute hypoxia elicit compartmentalization of cancer cells into symbiotic clusters. Hypoxic cancer cells metabolize glucose and secrete lactate; normoxic, vessel-proximal cancer cells import and metabolize the lactate in a process involving mTOR signaling. Co-inhibition of mTOR disrupts the symbiosis, in part by upregulating glucose transport in normoxic cells.

## Introduction

Judah Folkman’s vision of targeting the tumor neovasculature as a new modality of cancer therapeutics has inspired a series of drugs that either exclusively (e.g., bevacizumab) or primarily (e.g., sunitinib, axitinib, and sorafenib) inhibit VEGF signaling (McIntyre and Harris, 2015, Vasudev and Reynolds, 2014; and references therein) with associated beneficial responses, representing proof of principle and new additions to the armamentarium of anti-cancer drugs. However, as with many targeted therapies, clinical responses to angiogenesis inhibitors (AI) are typically limited, manifested as increased, but limited progression-free survival and variable (or no) overall survival (Vasudev and Reynolds, 2014; and references therein). Concurrent with such clinical investigations, a number of preclinical studies of AI in various mouse models of human cancer have revealed multiple forms of adaptive resistance that enable tumors to evade the effects of AI therapy (Bergers and Hanahan, 2008, Clarke and Hurwitz, 2013, Welti et al., 2013, McIntyre and Harris, 2015, Rigamonti et al., 2014, Rivera et al., 2015). One such model—*RIP1Tag2* transgenic mice, which develop de novo pancreatic neuroendocrine tumors (PanNET) via a multistage pathway—has been particularly instructive: tumorigenesis involves a discrete angiogenic switch (Folkman et al., 1989), which is necessary for initial tumor formation. AIs, in particular ones targeting the VEGF signaling pathway, show demonstrable efficacy in this model (Bergers et al., 2003), the results of which motivated clinical trials that led to the approval of one such agent, sunitinib, in human PanNET (Raymond et al., 2011). Importantly, however, AIs are not curative in this mouse model, much as in human cancer patients. The basis for treatment failure lies in part in the development of multiple forms of adaptive resistance to AIs, including revascularization mediated by alternative pro-angiogenic signaling circuits (Casanovas et al., 2005), recruitment of vascular-protective myeloid cells (Shojaei and Ferrara, 2008), and cooption of normal tissue vessels via increased invasion and metastasis (Sennino et al., 2012, Ebos and Kerbel, 2011, Ebos et al., 2009, Pàez-Ribes et al., 2009).

The RIP1Tag2 model has also been used to investigate other cancer hallmarks, including resistance to apoptosis and induction of invasiveness, which are variously regulated by signaling from receptor tyrosine kinases, including EGFR, IGFR, IR, and ALK (Chun et al., 2010, Nolan-Stevaux et al., 2010, Ulanet et al., 2010); these signals converge in part on the mTOR kinase, which orchestrates a broad program affecting cell survival and metabolism (Laplante and Sabatini, 2012, Cornu et al., 2013). Notably, both preclinical (Chiu et al., 2010) and clinical studies (Yao et al., 2011) have demonstrated the efficacy of targeting mTOR in PanNET.

We have continued to assess the effects on AI therapy in this PanNET model, including a comparative analysis of two multi-kinase inhibitors, sunitinib and axitinib, both of which target the VEGFR/PDGFR signaling pathways to inhibit new blood vessel growth and disrupt endothelial cells and pericytes of the preexisting tumor vasculature (Bergers et al., 2003), reducing vascular density and functionality rather than producing the vascularization characteristic of less potent AI such as bevacizumab. Although both compounds have a similar target profile, axitinib exhibits fewer off target effects and toxicity (Gunnarsson et al., 2015) (http://www.accessdata.fda.gov). In the course of further characterizing cellular and histologic responses to the two drugs, in particular their effects on the aforementioned mTOR signaling pathway, we observed an intriguing switch in the pattern of mTOR activity, reflected by phosphorylation of its downstream effector S6. Specifically, the diffuse pattern seen in untreated tumors was replaced by one of focal clusters. This focal reorganization of mTOR signaling motivated the investigation reported below, where we implicate metabolic symbiosis as another mode of adaptive resistance to anti-angiogenic therapy.

## Results

### Sunitinib/Axitinib Treatment Affects mTOR Signaling in Mouse PanNET

**Figure 1:**
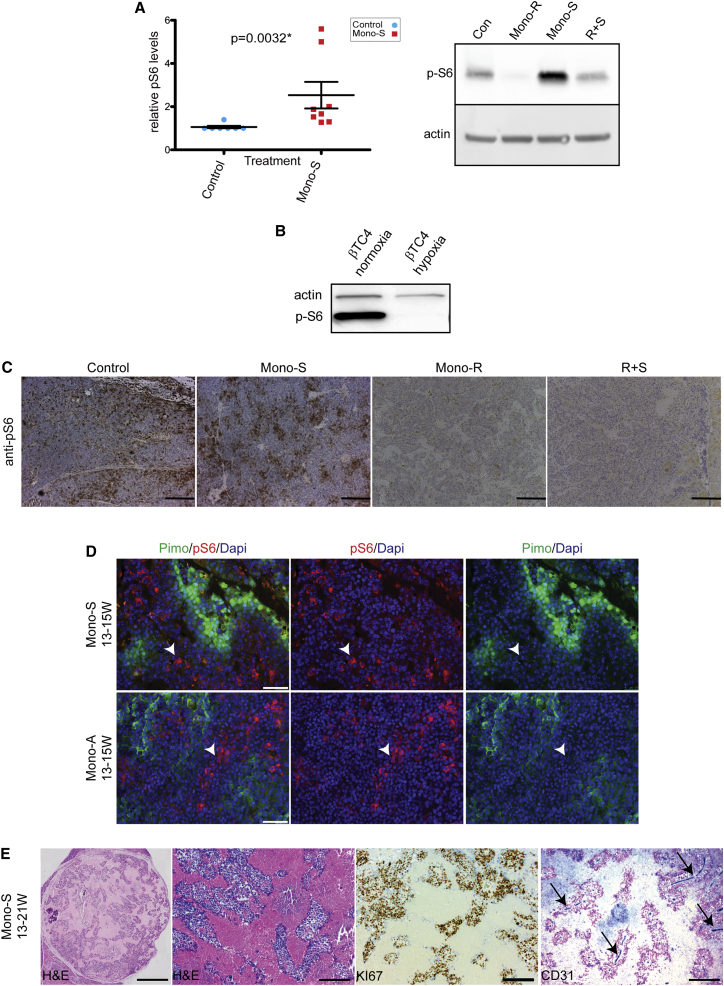
Effects of Potent Angiogenesis Inhibition on mTOR Signaling; Upregulation and Relocalization into Focal Clusters (A) Quantitation of p-S6 intensity in western blots (WB) of tumors treated with sunitinib versus sham control (top left). All samples were first normalized using a Bradford assay and a WB for actin. The blots were probed with p-S6 antibodies, and the relative values of Mono-S- (n = 8, red) treated tumors were compared to controls (blue, n = 7) by quantitation using the Fusion FX7 imaging system (see Supplemental Information). A similar trend was seen for tumors treated with axitinib (data not shown). The protein lysates were prepared from PanNET tumors collected from RIP1Tag2 mice, following a 1-week trial from 14–15 weeks of age. The mice were treated daily (see Supplemental Information) with vehicle control (Con), rapamycin monotherapy (Mono-R), sunitinib monotherapy (Mono-S), or a combination of the two (R+S) (top right). The lysates were normalized to actin by WB, blotted, and probed with anti-p-S6 as a readout for mTOR signaling and reprobed for actin normalization. (B) The βTC4 cell line was cultured in normoxic (20% O_2_/5% CO_2_) or hypoxic (1% O_2_/5% CO_2_) conditions, and the lysates were prepared and analyzed by WB as above. (C) Tissue sections from tumors treated for 4 weeks in an intervention trial (see Figure 4A for a description of trial formats) were used for IHC using anti-p-S6. The representative images are shown. The scale bars represent 100 μm. (D) Representative images from tissue sections of tumors treated with sunitinib (top) or axitinib (bottom). Pimonidazole (pimo, green) staining indicates tumor hypoxia (right), p-S6 reactivity (red, arrowheads) indicates that mTOR/p-S6 signaling is mostly excluded from the hypoxic regions (middle), and the merged images are shown in the left image. The scale bars represent 50 μm. (E) A highly regressed sunitinib-treated tumor after 8 weeks of Mono-S (13–21 weeks). The islands of highly proliferative cells (Ki-67+) are embedded in fibrotic tissue, which are organized around the few remaining blood vessels (CD31, blue, arrows). The scale bar represents 700 μm in the leftmost image and 200 μm in all others.

Given that mTOR signaling is prominently involved in the PanNET phenotype (see above), we had asked whether it was affected by disruption of the tumor vasculature with sunitinib. Indeed, mTOR signaling was found to be elevated in a set of sunitinib-treated tumors, as reflected in the heightened levels of phospho-S6 kinase (p-S6). In contrast, sunitinib treatment of cultured mouse PanNET cells did not elicit this upregulation (data not shown). To substantiate this observation, molecular efficacy trials were performed with sunitinib, in which mice with late stage tumors were treated for 7 days followed by analysis of tumor lysates by western blotting. Sunitinib elicited an increase in p-S6 (Figure 1A, left), which was blocked by concomitant administration of the mTOR inhibitor rapamycin (Figure 1A, right), demonstrating that its upregulation is mTOR dependent. Axitinib-treated tumors produced a similar trend (data not shown).

The elevated mTOR signaling was surprising in that sunitinib- and axitinib-treated tumors become hypoxic in response to collapse of the tumor vasculature (Pàez-Ribes et al., 2009, Sennino et al., 2012), and observations presented below), and mTOR is typically downregulated in hypoxic conditions (Brugarolas et al., 2004). Indeed, a derivative PanNET cancer cell line (βTC4) downregulated p-S6 in hypoxic versus normoxic conditions (Figure 1B). Intrigued by this apparent dichotomy, we further investigated mTOR signaling in tumors treated for 4 weeks using immunohistochemistry. As shown in Figure 1C, p-S6 was elevated in sunitinib-treated tumors, but its expression was relocalized from the diffuse pattern seen in control tumors into focal clusters. The specificity of the p-S6 staining in control and sunitinib-treated tumors was confirmed by treatment with rapamycin, which ablated p-S6 expression, even when dosed in combination with sunitinib.

Next, we assessed the relationship between p-S6 expression and hypoxia in treated tumors. Co-staining of sunitinib- and axitinib-treated tumors with antibodies to p-S6 and pimonidazole—to reveal regions of hypoxia—revealed an anti-correlation: p-S6 was predominantly expressed in the normoxic, and not hypoxic, regions of the treated tumors (Figure 1D).

Eventually, as the vascular regression and inhibition of new growth continues, the normoxic regions seen in Figure 1D can coalesce into the focal clusters seen in Figure 1C. This is further illustrated in Figure 1E, which shows a highly necrotic/hypoxic tumor (left); within the regions of fibrosis/necrosis, focal clusters of viable cancer cells can be seen (H&E, middle left). These clusters are highly proliferative (Ki-67, middle right) and are cuffing blood vessels (CD31, right).

### AI Treatment of Mouse PanNETs Produces Upregulation of Glucose and Lactate Transporters in Patterns Suggestive of Metabolic Symbiosis

**Figure 2:**
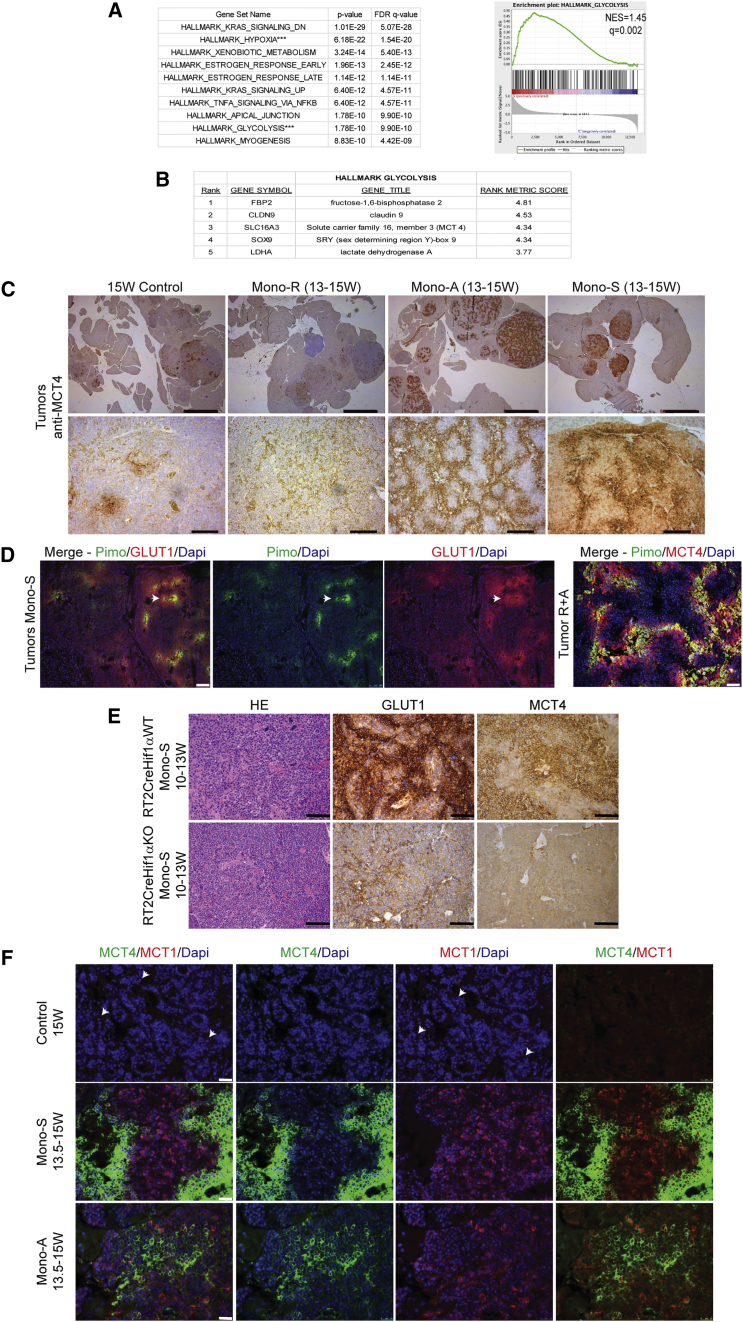
Gene Specific Expression Analysis Reveals a Strongly Glycolytic Signature in Mono-S-Treated Tumors (A) Gene set overlapping analysis on the top 2,500 upregulated genes in sunitinib treatment compared to vehicle treated controls, revealing that glycolysis and hypoxia were among the most enriched pathway signatures (left). The glycolysis enrichment plot for sunitinib-treated tumors, including the profile of running ES Score are shown (right). (B) Top five ranked genes from the hallmark glycolysis gene set, adopted from the Molecular Signatures Database of the Broad Institute, for the sunitinib-treated cohort. (C) MCT4 expression was assessed using IHC on 15 week control tumors (Con, left) and tumors treated with rapamycin monotherapy (Mono-R, middle left), axitinib (Mono-A, middle right), or sunitinib (Mono-S, right). The MCT4 expression is high in 4/4 larger and 5/6 smaller Mono-A- and 4/4 larger Mono-S-treated tumors and absent in Con and Mono-R-treated tumors. The scale bars represent 2 mm in top image and 200 μm in the bottom image. (D) Pimonidazole (pimo, green) staining was performed to assess tumor hypoxia (center left); the glucose transporter, GLUT1, is shown in red (center right), and the leftmost image depicts the merged images. A merged image of R+A-treated tumors for MCT4 and pimonidazole is depicted in the rightmost image. The GLUT1 and MCT4 staining is highest in the most pimonidazole+/hypoxic regions, but can also be found in the peri-hypoxic areas. The scale bars represent 100 μm in the three left images and 50 μm in the right image. (E) IHC using anti-GLUT1 (middle) and anti-MCT4 (right) indicates that their expression is highly reduced/absent in tumors containing a cell-type-specific (β-cell) gene knock out of HIF1α that were treated with sunitinib. The top row shows a representative Rip1Tag2_Rip1Cre_Hif1αWT tumor, while the bottom row shows a Rip1Tag2_Rip1Cre_Hif1αfl/fl littermate whose tumors do not express Hif1α; this result is representative of all tumors from 4/4 wild-type (WT) versus 6/6 HIF1α KO mice, all similarly treated with sunitinib. The scale bar represents 100 μm. (F) Monotherapy with sunitinib (middle row) or axitinib (bottom row) elicits upregulation of MCT4 (green, first, second, and fourth) versus control untreated tumors (top row); in addition, MCT1 (in red) is upregulated in both AI-treated arms (middle and bottom rows, first, third, and fourth) versus controls (top row, first, third, and fourth). The scale bar represents 25 μm.

The clustering of viable cancer cells around the few remaining blood vessels inside long-term treated tumors is consistent with their need for oxygen, but also for blood-borne nutrients, in particular glucose. We performed short-term trials using sunitinib alone or in combination with rapamycin and then performed RNA-sequencing (seq) analysis on the treated and control tumors. We conducted enrichment analysis on the top 2,500 unregulated genes in the control versus treated tumors, and the top ten enriched hallmark pathways for sunitinib monotherapy are depicted in Figure 2A, including hypoxia and glycolysis. The enrichment plot for the hallmark glycolysis signature (Figure 2A) includes *Slc16a3* (MCT 4) and *Ldha* (lactate dehydrogenase A; Figure 2B), along with (to a lesser degree) *Slc2a1* (GLUT1, data not shown). Both sunitinib and axitinib produced marked reductions in tumor vasculature (Figure S1A) with widespread hypoxia, consistent with the hypoxia signature. Motivated by the glycolysis signature and the known induction of glycolysis by hypoxia, we assessed expression by immunostaining for the lactate transporter MCT4 and the glucose transporter GLUT1, whose elevated co-expression is diagnostic of glycolysis (Ullah et al., 2006). Concordantly, the striking upregulation of *Slc16a3*/MCT4 revealed by the RNA profiling of sunitinib-treated tumors was detected by immunohistochemistry in tissue sections from the majority of sunitinib- and axitinib-treated tumors and was very low/absent in control or rapamycin-treated tumors (Figure 2C). In treated tumors, GLUT1 and MCT4 were upregulated in the hypoxic regions, as revealed by co-staining with pimonidazole (Figure 2D). Such upregulation in hypoxic conditions is consistent with previous reports that GLUT1 and MCT4 are regulated by the HIF hypoxia response system (Ebert et al., 1995, Ullah et al., 2006, Seagroves et al., 2001). To confirm this interpretation, we evaluated compound mice carrying a tissue-specific gene knock out of HIF1α in the oncogene-expressing cancer cells. Indeed, sunitinib treatment failed to upregulate GLUT1 and MCT4 in the Hif1α-knockout (KO) tumors (Figure 2E), supporting the expectation that HIF1α drives their upregulation.

The compartmental expression of GLUT1/MCT4 was reminiscent of a phenomenon—observed in certain tumors—known as metabolic symbiosis, wherein hypoxic regions import and metabolize glucose (Ebert et al., 1995) and secrete lactate, while normoxic regions import and metabolize the lactate (Sonveaux et al., 2008, Kennedy et al., 2013). We therefore assessed expression of the lactate transporter MCT1 and found that it was upregulated in a distinct compartment from the hypoxic, MCT4 positive region (Figure 2F), although a small minority of cells are MCT1^+^/MCT4^+^ double positive (Figure S1B).

### Mouse PanNET Tumors and Cell Lines Consume and Catabolize Lactate to Establish Metabolic Symbiosis

**Figure 3:**
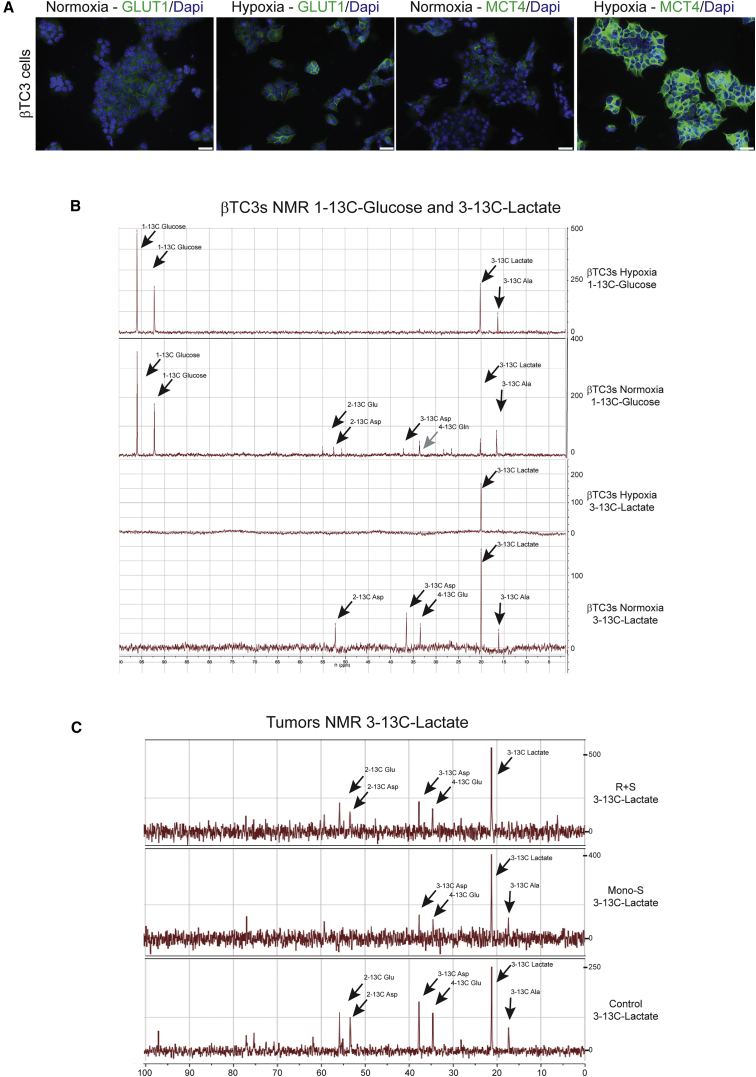
Mouse PNET Cell Lines and Tumors Consume and Catabolize Lactate to Establish Metabolic Symbiosis (A) βTC3 cancer cells cultured in normoxic conditions show low levels of GLUT1 (green) and MCT4 (green) expression, whereas both proteins are upregulated in hypoxic conditions. The scale bars represent 25 μm. (B) NMR analysis of βTC3 cells, cultured in low glucose (6.25 mM glucose + 2 mM Gln + FCS), plated in the same media at 7 × 10^6^ for 2 days, switched to 0% glucose/0% Gln + FCS ON, then plated in 20 mM ^1^^–^^13^C-glucose + 2 mM Gln +FCS (top) or 20 mM ^3^^–^^13^C-lactate + 2 mM Gln+ FCS, cultured 16 hr in hypoxic (1% O_2_/5% CO_2_) or normoxic conditions (20% O_2_/5% CO_2_), and harvested for NMR. The net production of lactate in CM from time 0 in ^1^^–^^13^C-glucose conditions in hypoxia was +15.6 mM and normoxia +4.6 mM, while ^3^^–^^13^C-lactate levels were either increased in hypoxia by +0.4 mM or reduced in normoxia by −2.7 mM. The gray arrow highlights ^4^^–^^13^C-glutamine, which was found as a product of ^3^^–^^13^C-lactate catabolism in βTC3 cells when these results were replicated (data not shown). (C) Tumor-bearing mice were treated for 10 days with Mono-S or R+S, or vehicle control, and then infused with ^3^^–^^13^-C-lactate for 90 min prior to sacrifice. The tumors were excised, quick frozen in liquid nitrogen, and prepared for NMR (see Experimental Procedures).

The hypothesis of metabolic symbiosis demands that the secreted lactate is not merely “toxic acidic waste”, but rather a source of fuel that is imported and metabolized by the normoxic cancer cells. We assessed whether cultured βTC3 cells could recapitulate key aspects of the hypoxic response in culture and found that GLUT1 and MCT4 were upregulated under hypoxic conditions (Figure 3A). We further assessed lactate utilization in cultured cancer cells and in tumor-bearing mice, by supplying isotopically labeled lactate or glucose, and assessing their catabolism into metabolites by NMR spectroscopy (Figures 3B and 3C). Thus, βTC3 cancer cells were cultured in hypoxic and normoxic conditions, in which they were supplied with ^1^^–^^13^C-glucose or with ^3^^–^^13^C-lactate in glucose-free media, in both cases similarly supplemented with glutamine and fetal calf serum (FCS); after 20 hr, the cells were harvested for NMR analysis. At this time point, cells cultured in ^1^^–^^13^C-glucose increased the levels of lactate in conditioned media both in hypoxia and normoxia, whereas cells cultured in ^3^^–^^13^C-lactate reduced the levels of lactate in conditioned media under normoxia and slightly increased lactate in hypoxic conditions (Figure 3B, legend). The ^13^C NMR analysis revealed that in aerobic conditions ^3^^–^^13^C-lactate was catabolized to C4-glutamate, C2- and C3-aspartate (Asp), and C3-alanine (Figure 3B), consistent with previous studies on lactate metabolism by cancer cells and neurons (Kennedy et al., 2013, Waagepetersen et al., 1998, Yang et al., 2014). In contrast, lactate was imported, but not catabolized in hypoxic conditions (Figure 3B). Notably, the lactate catabolites coincide with those produced by ^1^^–^^13^C-glucose, indicating that both sets are likely produced through the same intermediate, pyruvate.

Next, tumor-bearing mice treated for 10 days with sunitinib, rapamycin+sunitinib, or vehicle control were infused with ^3^^–^^13^C-lactate and the tumors excised and evaluated by NMR. Both treated and control tumors metabolized lactate (Figure 3C). Additionally, the branched metabolic pathways involved in lactate catabolism may be altered in activity. The prominent ^3^^–^^13^C-alanine band produced from ^3^^–^^13^C-lactate → ^3^^–^^13^C-pyruvate + glutamate → ^3^^–^^13^C-Ala + α-ketoglutarate is relatively undiminished versus the glutamate and Asp species in the sunitinib-treated tumors; notably, the pathway producing ^3^^–^^13^C-Ala + α-ketoglutarate is involved in sustaining tricarboxylic acid (TCA) cycle intermediates important both for energy production and biosynthesis of cellular building blocks, a process referred to as anaplerosis. Intriguingly, RNaseq and RT-quantitative (q)PCR analysis (Table S1) of sunitinib versus control tumors revealed upregulation of *Gpt2*, an aminotransferase catalyzing the transamination that produces alanine + α-ketoglutarate from pyruvate and glutamate. In addition, *Gls*2, which converts glutamine to glutamate in the first step of its metabolism, is also upregulated (Table S1). In contrast, the rapamycin+sunitinib-treated tumors did not produce ^3^^–^^13^C-Ala. Rather, lactate-derived pyruvate evidently was converted to acetyl-CoA to enter the TCA cycle, producing C2- and C4-glutamate and C2- and C3-Asp (Figure 3C). Thus, the data suggest that glutamine-associated catabolism of lactate to alanine becomes favored in the context of sunitinib therapy, which in the combination-treated tumors is repressed—directly or indirectly—by mTOR inhibition. Collectively, these observations, along with previous studies indicating the link between glutamine metabolism and mTOR signaling (Csibi et al., 2014, Durán et al., 2012), led us to consider the possible involvement of glutamine and the observed metabolism of lactate in the induction of mTOR signaling.

### mTOR Signaling in Cultured Cancer Cells can Be Upregulated by Lactate and Glutamine Metabolism

**Figure 4:**
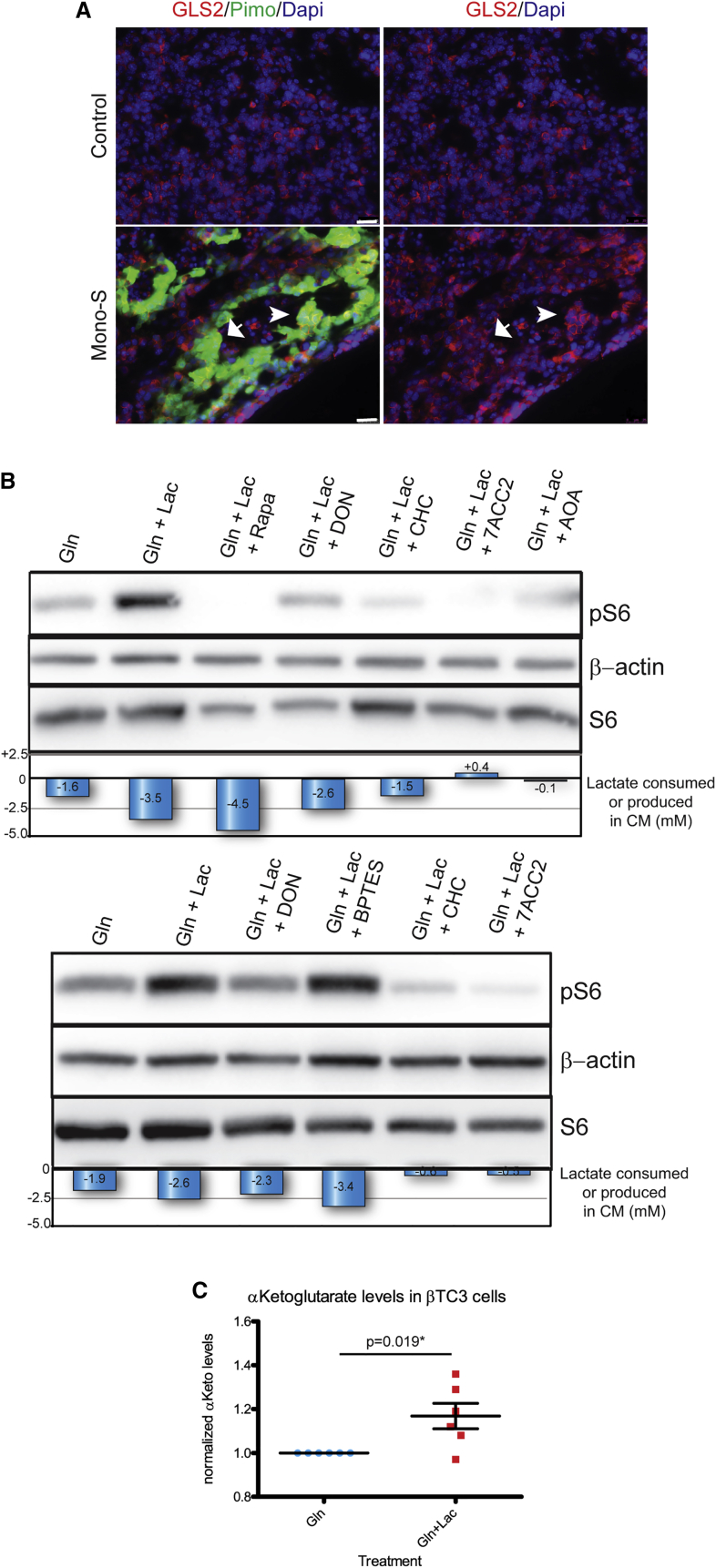
Metabolic Regulation of mTOR Signaling (A) Pimonidazole (green) is merged with anti-GLS2 (red) in left images, and right images depict tumors anti-GLS2 only (red) in control untreated tumors (top) and sunitinib-treated tumors (bottom). The arrow indicates GLS2-negative staining, while the arrowhead indicates GLS2-positive staining in hypoxic cells. The scale bars represent 25 μm. (B) βTC3 cells, acclimated to low glucose as in Figure 3, were cultured in 2 mM glutamine (Gln), or 2 mM Gln + 20 mM lactate ± selective metabolic inhibitors, and assessed for p-S6 levels. Both βTC3s and lactate avid SiHa cells (Figure S2C) markedly upregulated p-S6 when cultured in lactate + Gln versus Gln alone; this upregulation could be reversed by 100 nM rapamycin treatment, or partially reversed using 40 μM DON, a competitive inhibitor of glutaminase. In addition, blocking lactate uptake with the MCT1 inhibitors 1 mM CHC or 10 μM 7ACC2 also reversed this upregulation, as did 200 μM AOA, which blocks the transamination of pyruvate + glutamate to alanine + α-ketoglutarate. The bottom images depict experiments performed with the selective GLS1 inhibitor, 50 μM BPTES, which failed to reverse the lactate induced pS6 upregulation, in contrast to DON, CHC, or 7ACC2. Below the blot is a graphic depicting the net consumption or production of lactate from time = 0. (C) Graphic depicting the relative production of α-ketoglutarate in Gln + lactate versus Gln-only conditions. The samples were normalized by protein concentration.

Motivated by the observation that expression of *Gls2* was increased in sunitinib-treated tumors (Table S1), we asked whether it was expressed in the hypoxic or normoxic compartment. Analysis of sunitinib-treated and control untreated tumors by fluorescent staining with anti-glutaminase 2 (GLS2) and pimonidazole demonstrated elevated expression of glutaminase 2 in both cases, and it could be found in both normoxic and hypoxic compartments in sunitinib-treated tumors (Figure 4A), a result that was substantiated via RT-qPCR analysis of βTC3s cultured under normoxic and hypoxic growth conditions (Table S2); we also analyzed additional metabolic genes for differential expression and found significant upregulation of *Slc2a1*, *Slc16a3*, and *Ldha* in hypoxic conditions, whereas *Slc2a2*, *Ldhb*, and *Gpt2* were elevated in normoxic growth conditions (Table S2).

Next, we investigated possible links between glutamine, lactate metabolism, and mTOR signaling. First, we assessed the importance of glutamine for cell proliferation of βTC3 cells by EdU incorporation, which revealed minimal difference between the various culture conditions containing glutamine, with or without glucose and/or lactate; there was, however, a block in cell-cycle progression under glucose without glutamine conditions (w/wo lactate) (Figure S2A), highlighting the dependence of these cells on glutamine. Then, we evaluated mTOR signaling in βTC3 cells (Figure 4B) cultured in different combinations of lactate and glutamine. Cells were cultured in FCS and glutamine-supplemented, glucose-free media, with or without lactate. Net changes in lactate levels in conditioned media (CM) were assessed. Lactate levels in glutamine-supplemented, glucose-free cell media decreased reproducibly under aerobic conditions, consistent with its uptake by βTC3 cells (Figures 4B and S2B). Moreover, there was significant upregulation of p-S6 levels in cells cultured in lactate + glutamine versus glutamine alone (Figure 4B, top and bottom), correlating with a net reduction of lactate in CM. As expected, upregulation of p-S6 could be reversed by rapamycin. Notably, p-S6 upregulation could also be reversed by inhibiting lactate uptake with CHC or 7ACC2, two distinctive inhibitors of the MCT1 lactate transporter (Sonveaux et al., 2008, Draoui et al., 2014) (Figure 4B, top and bottom). In addition, a glutaminase inhibitor, DON (Durán et al., 2012) (Figure 4B, top and bottom), and an alanine aminotransferase inhibitor, AOA (Lamonte et al., 2013) (Figure 4B, top), also reversed p-S6 upregulation, whereas a glutaminase 1-specific inhibitor, BPTES, did not (Figure 4B, bottom). We observed similar effects of lactate and glutamine on mTOR signaling in lactate-avid SiHa ovarian cancer cells (Figure S2C), which have been extensively used to study metabolic symbiosis (Sonveaux et al., 2008). Collectively, the data support a mechanism whereby cancer cells take up and metabolize lactate in the context of bioavailable glutamine in normoxic, but not hypoxic conditions (Figures 3B and 3C), thereby upregulating mTOR signaling (Figure 4B). A schematic depicting the branched metabolic pathways involved in lactate catabolism in control and treated Rip1Tag2 PanNET tumors is depicted in Figure S2D. In addition, we found that βTC3 cells cultured in lactate + glutamine upregulated α-ketoglutarate compared to cells cultured in glutamine only (Figure 4C). Concordantly, Sonveaux and colleagues recently reported a link between lactate and glutamine metabolism in cultured SiHa and HeLa cancer cells under oxidative conditions (Pérez-Escuredo et al., 2016).

### Effects of Co-targeting Angiogenesis and mTOR Signaling

**Figure 5:**
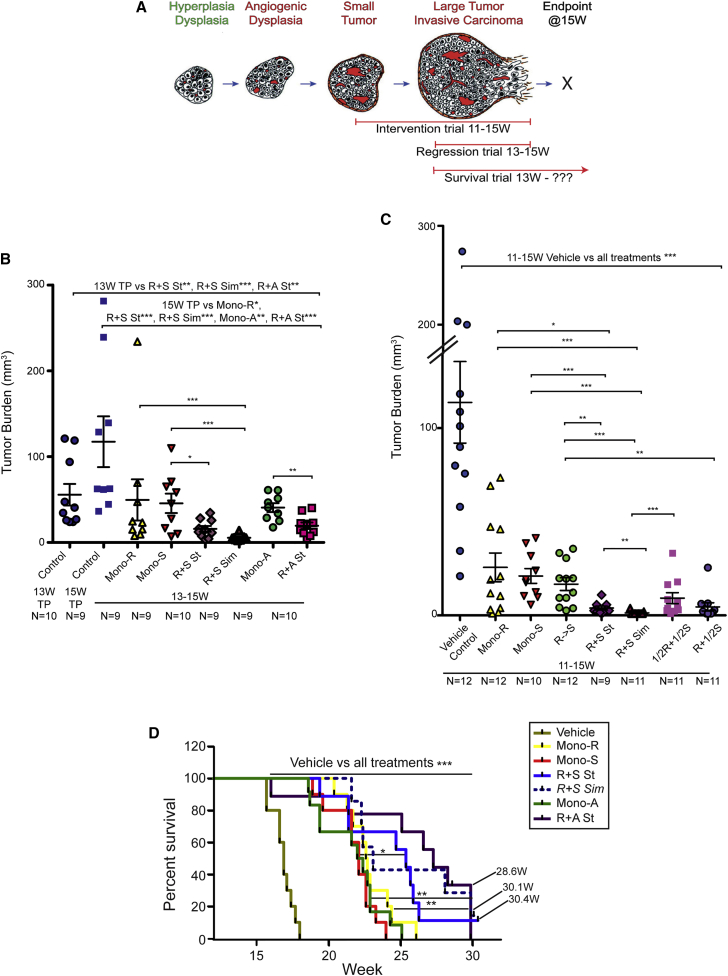
Therapeutic Targeting of AI-Induced, p-S6+ Clusters with Rapamycin (A) Trial designs are indicated in this schematic: trials were initiated and terminated at discrete time points, as established by previous studies in the Rip1Tag2 model (Bergers et al., 1999). The intervention trials were initiated at 11 weeks when tumors were small (Figure 5C), while fixed endpoint regression trials (Figure 5B) and open endpoint survival trials (Figure 5D) were initiated at 13 weeks when tumors were already large; molecular efficacy trials commenced at 13.5–14 weeks and proceeded for 7–10 days. (B) Fixed endpoint regression trials were performed from 13–15 weeks, as described in the Supplemental Information. Mean values ± SEM are indicated, and a two-tailed Mann-Whitney test was used to assess statistical significance; 13 week timepoint (TP) control versus R+S St and R+A St, p = 0.003^∗∗^ and versus R+S Sim, p = 0.0003^∗∗∗^; 15 week TP control versus Mono-R, p = 0.02^∗^, R+S St, p = 0.0003^∗∗∗^, R+S Sim, p = 0.0004^∗∗∗^, Mono-A, p = 0.006^∗∗^, and R+A St, p = 0.0005^∗∗∗^; Mono-R versus R+S Sim, p = 0.0008^∗∗∗^; Mono-S versus R+S St, p = 0.044^∗^ and R+S Sim, p = 0.0008^∗∗∗^; and Mono-A versus R+A, p = 0.004^∗∗^ (^∗^p = 0.05–0.01, ^∗∗^p = 0.009–0.001, and ^∗∗∗^p < 0.001). (C) Fixed endpoint intervention trials were performed for 4 weeks, commencing at 11 weeks, and described in Supplemental Information. Mean values ± SEM are indicated. Two-tailed Mann-Whitney test for statistical significance; 11–15 week vehicle control versus Mono-R and Mono-S, p = 0.0004^∗∗∗^, R≥S and R+S St, p = 0.0001^∗∗∗^, R+S Sim, p = 0^∗∗∗^, and 1/2R+1/2S and R+1/2S, p = 8 × 10^−5^; Mono-R versus R+S St, p = 0.036^∗^ and R+S Sim, p = 0.0006^∗∗∗^; Mono-S versus R+S St, p = 0.0009^∗∗∗^ and R+S Sim, p = 0.0001^∗∗∗^; R->S versus R+S St, p = 0.0032^∗∗^ and R+S Sim, p = 8 × 10^−5^^∗∗∗^ and R+1/2S (p = 0.0023^∗∗^); R+S St versus R+S Sim, p = 0.002^∗∗^; and 1/2R+1/2S versus R+S Sim, p = 0.0009^∗∗∗^ (^∗^p = 0.05–0.01, ^∗∗^p = 0.009–0.001, and ^∗∗∗^p < 0.001). (D) Open endpoint survival trials were performed in Rip1Tag2 mice from 13 weeks of age until animals became moribund and were sacrificed. Cohorts were composed of 7–12 mice/arm, and p values were derived using the log rank test (LR). Survival was assessed for each cohort as mean survival in weeks ± SD: control vehicle-treated mice (n = 10; 16.9 ± 0.8 weeks); Mono-S (n = 10; 21.8 ± 1.6 weeks); Mono-R (n = 10; 22.9 ± 1.7 weeks); Mono-A (n = 12; 21.7 ± 2.2 weeks); and R+S St (n = 9; 24.51 ± 3.3 weeks), R+S Sim (n = 7; 25.4 ± 3.8 weeks), and R+A St (n = 9; 25.8 ± 4.4 weeks). The R+S Sim arm was comprised of fewer animals than the other arms, signified by a stippled line, and was not statistically different than each cognate monotherapy. Long surviving, healthy mice from different arms were sacrificed for evaluation of TB and metastasis at the following time points: R+S St, 30.4 weeks; R+S Sim, 30.1 weeks; and two mice from R+A St, 28.4 weeks. All of the treated arms show significantly higher survival than the vehicle-treated mice of p < 0.0001^∗∗∗^, except for Mono-A, which has a p < 0.0002^∗∗∗^. R+S St versus Mono-S, p = 0.015^∗^; R+A St versus Mono-A, p = 0.002^∗∗^; and Mono-R, p = 0.004^∗∗^ (^∗^p = 0.05–0.01, ^∗∗^p = 0.009–0.001, and ^∗∗∗^p < 0.001). See also Figures S3A and S3B.

In light of the proposition that potent AIs were inducing tumors to adapt a strategy of metabolic symbiosis, and the observed relocalization of mTOR signaling into the normoxic compartment of putative symbiotic clusters, we investigated the impact of blocking mTOR signaling on the AI-induced symbiosis and consequent tumor phenotypes. Regression trials (depicted in Figure 5A) were initiated at 13 weeks, when the mice already had appreciable tumor burden, and continued to a defined endpoint 2 weeks later (15 weeks), when most untreated animals have succumbed to hypoglycemia attributable to increased insulin secretion from the multiple pancreatic tumors that develop. Reasoning that vascular collapse might affect the bioavailability of rapamycin in the tumor microenvironment, we tested a trial regimen in which R+S and R+A arms were dosed for an initial 2D with rapamycin alone, followed by continuous dosing in combination with the AI (called “stagger” or “St”). Another cohort of R+S was dosed simultaneously with both drugs from the beginning (“simultaneous” or “Sim”). All three monotherapies (Mono-S, red triangles; Mono-R, yellow triangles; and Mono-A, green circles) showed anti-tumor activity, blocking further growth to produce tumor stasis compared to 13 week controls (Figure 5B, blue circles). In marked contrast, each of the combinations of rapamycin plus an AI (R+S-St, purple diamonds; R+S-Sim, purple triangles; and R+A-St, rose squares) produced significant tumor regression versus the 13 week starting time point (Figure 5B, blue circles). An additional 4-week-long intervention trial was performed with sunitinib, starting at 11 weeks, when the tumors were smaller, until the same defined 15 week endpoint (Figure 5C). Again, while Mono-S (red triangles) and Mono-R (yellow triangles) produced significantly smaller tumors than vehicle-treated controls (blue circles), their combination (R+S-St, purple diamonds and R+S-Sim, purple triangles) produced significantly lower tumor burden than either efficacious monotherapy. Other dosing strategies were also assessed, including a shift from Mono-R to Mono-S (Figure 5C, R->S, green circles), which appeared indistinguishable from each monotherapy in regard to tumor burden (TB). There was also an efficacious response to the combination R+S when sunitinib was reduced by half (Figure 5C, R+1/2S, blue-gray circles). Thus, both experimental therapeutic trials demonstrated anti-tumoral efficacy for the combination of the mTOR inhibitor rapamycin and a potent AI.

In order to further characterize the therapeutic response, open endpoint survival trials were performed with the combinations, in comparison to monotherapy- and vehicle-treated controls. Cohorts of mice were treated starting at 13 weeks until they became moribund and were sacrificed (Figure 5D). All treatments, including the monotherapy arms, led to a significant survival advantage versus vehicle-treated mice. End stage tumors had progressed to a considerable size on rapamycin monotherapy, larger on average than untreated controls, indicating an adaptive resistance involving enhanced growth rates (Figure S3A), as reported previously (Chiu et al., 2010). Overall, TB at end stage was higher in regimens involving axitinib versus sunitinib (Figure S3A). Interestingly, both AIs produced tumors that never revascularized (data not shown). Additionally, the combination therapies reduced the frequency of metastasis compared to both AI monotherapies, despite the significantly longer survival of mice in the dual therapy arms (Figure S3B). It is unclear why the mice with only residual TB were dying, which is a topic worthy of future investigation.

### mTOR Inhibition Disrupts Metabolic Symbiosis

The combination of rapamycin plus sunitinib treatment for 1–2 weeks reproducibly elicited tumor regression, characterized by widespread necrosis and fibrosis, and markedly reduced proliferation, particularly in large tumors (Figures S4A, S4B, and S6). In contrast, while 8 weeks of Mono-S also produced tumors with pronounced necrosis and fibrosis, the remaining tumor cells were highly proliferative (Figure 1E). When 13- to 15-week-treated tumors were analyzed comparatively for proliferation rates via Ki-67 staining, there was a significant reduction in proliferation in R+S-treated tumors versus controls and both monotherapies (Figure S4B). Tumors treated with Mono-R had limited necrosis (data not shown), consistent with this and our previous study indicating it elicited heighted rates of apoptosis without appreciable necrosis (Chiu et al., 2010) (Figure S4C).

**Figure 6:**
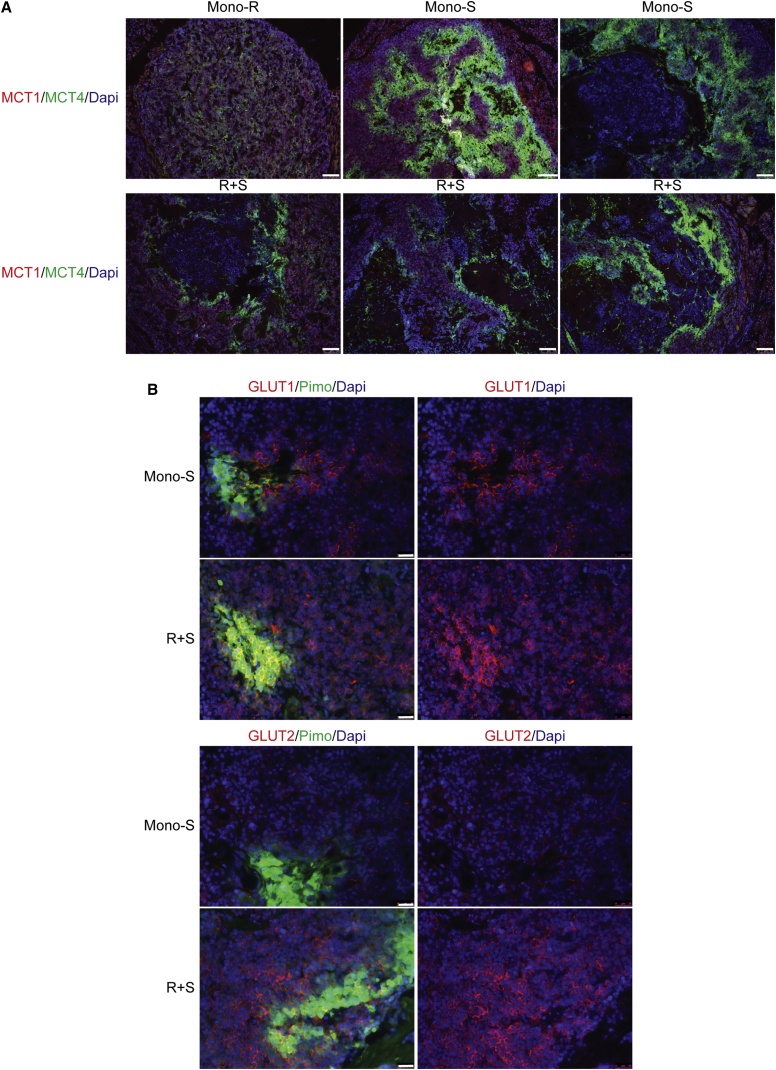
mTOR Inhibition Disrupts Metabolic Symbiosis (A) Evaluation by tissue immunostaining of MCT1 (red) and MCT4 (green) expression in representative tumors treated with Mono-R or Mono-S (top row) or with the combination (bottom row). The Mono-R-treated tumors (top left) appear similar to control vehicle-treated tumors (Figures 2F and S7) in having relatively weak expression of the MCT1/4 transporters. In the R+S-treated tumors (bottom), MCT4-expressing regions (green) are reduced versus MCT1-expressing regions, which have expanded (red), in contrast, note relatively more expanded MCT4-positive regions in Mono-S-treated tumors (top: center and right: green). The scale bar represents 100 μm. See also Figure S7. (B) In contrast to the overlapping distribution between GLUT1 (red) and hypoxia (pimo, green) in both Mono-S and R+S-treated tumors (top row), GLUT2 (red) is selectively upregulated in the non-hypoxic compartment of R+S-treated tumors (bottom row). The scale bars represent 25 μm. See also Figure S5B.

Seeking to understand the basis for this heightened necrosis, and having identified putative symbiotic clusters of cancer cells associated with remaining blood vessels in the Mono-S-treated tumors, we asked whether rapamycin was affecting symbiosis in the R+S-treated tumors. The clusters could still be detected, as revealed by co-staining with MCT1 and MCT4, but their morphology was altered. Surprisingly, we observed that cancer cells in the hypoxic GLUT1^high^, MCT4^high^, and mTOR^low^ compartment were selectively depleted compared to those cells in the normoxic GLUT1^low^, MCT1^high^, and mTOR^high^ regions where rapamycin was acting to suppress mTOR signaling. Only a rim of MCT4 cells could be detected, flanking regions of necrosis, in contrast to the multiple layers of such cells seen in the Mono-S-treated tumors (Figure 6A). In contrast, rapamycin did not obviously disrupt the (previously p-S6^high^) MCT1^high^ compartment (Figure 6A). Thus, inhibition of mTOR signaling in the normoxic compartment was disrupting the symbiosis, but via an unexpected paracrine mechanism.

A tenant of the symbiosis hypothesis is that the normoxic cancer cells have not upregulated glucose transporters, in contrast to the hypoxic cells (Figure 2D), thereby sparing limited bioavailable glucose to diffuse from the nearest blood vessel to distal, but still viable hypoxic cancer cells. We wondered, therefore, if mTOR inhibition affected expression of glucose transporters? We first surveyed untreated tumors and observed heterogeneous expression of GLUT1 and a related transporter GLUT2, which is involved in glucose homeostasis in pancreatic islets (Guillam et al., 1997) (Figure S5A). A third transporter, GLUT3, was not consistently expressed (data not shown). Mono-S- and R+S-treated tumors were stained with antibodies that recognized pimonidazole adducts, indicative of hypoxia, and anti-GLUT1 and anti-GLUT2 antibodies (Figure 6B). The analysis revealed appreciable upregulation of GLUT2 expression (red staining) in the normoxic regions of R+S, but not Mono-S-treated tumors (Figure 6B, bottom); the Mono-S tumors express very little GLUT2 (or GLUT1) in normoxic regions proximal to the vessels. In contrast to GLUT2, GLUT1 is not widely upregulated in the normoxic compartment of R+S-treated tumors. We also performed comparative IHC using automated staining with GLUT1 and GLUT2 and saw a similar upregulation of GLUT2 in the normoxic regions of R+S- versus Mono-S-treated tumors (Figure S5B). Western blot analysis on tumors also confirmed GLUT2 upregulation in R+S-treated tumors versus Mono-S (Figure S5C), as did qRT-PCR (Table S1). There was also a modest upregulation of GLUT2 in βTC3 cells cultured in glutamine+lactate+rapamycin versus glutamine ± lactate (Figure S5D), conditions we believe may be a surrogate for those found in the oxidative cancer cells inside AI-treated tumors. Although consistent with the in vivo results, further work is required to substantiate these findings and determine the molecular pathway that governs this regulation. Thus, we envision that the normoxic compartment of the putative symbiotic clusters switches, in the context of mTOR inhibition, to importing and metabolizing blood-borne glucose instead of sparing it to favor lactate. Consequently, the vascular non-proximal hypoxic cells would starve from glucose deprivation and die; likely exacerbated by lactic acidosis, since the normoxic cells are now net secretors of lactate as a consequence of aerobic glycolysis. Transmission electron microscopy (Figure S6) of cancer cells in the peri-necrotic region of a R+S-treated tumor reveals cells with granulated cytoplasm and grossly distended mitochondria, and abundant insulin granules, potentially indicative of the extreme conditions of insufficient oxygen and glucose, and an acidic microenvironment. To further substantiate these associations, Figure S7 shows images of additional Control, Mono-S-, and R+S-treated tumors on serial sections stained with anti-MCT1, anti-MCT4, and pimonidazole, matched with anti-phospho-S6 ribosomal protein (pS6), -GLUT1, and -GLUT2. These images further document the upregulation of MCT1/4 in Mono-S- and R+S-treated tumors versus untreated controls and abundant MCT1 staining in the R+S tumors. The pimonidazole/pS6 panels show strong and relatively homogenous staining of pS6 in non-hypoxic control tumors, and compartmentalized pS6 staining in the normoxic regions of Mono-S tumors, but no reactivity in the R+S-treated tumors. The TEM and immunostaining analysis both support the interpretation that the hypoxic compartment is being eliminated faster than the normoxic one in the R+S-treated tumors.

## Discussion

In this and the two companion reports in this issue of *Cell Reports* from Pisarsky et al., 2016 and Jiménez-Valerio et al., 2016, we describe an unanticipated new mode of adaptive resistance—metabolic symbiosis—that is induced in response to potent anti-angiogenic therapies that cause vascular collapse and consequent hypoxia. We show that cancer cells compartmentalize themselves in the acute condition of vascular insufficiency into comparatively hypoxic and normoxic compartments, based on their relative proximity to the few remaining functional blood vessels. The hypoxic cancer cells induce expression of the glucose importer GLUT1 and the lactate exporter MCT4, which is dependent on HIF1α. The normoxic cells express the lactate transporter MCT1, and we show that both normoxic cancer cells in culture and tumors in vivo import and catabolize lactate; thus lactate is not just toxic waste from glycolysis, but rather is being used for energy metabolism. Moreover, our data reveal that the metabolic branch of lactate catabolism involving glutamine metabolism is favored, and this branch is blocked in tumors treated with rapamycin and anti-angiogenic therapy. Moreover, PanNET cancer cells cultured in the presence of lactate and glutamine upregulate phospho-S6 ribosomal protein (p-S6); the proposition that glutamine metabolism upregulates mTOR activity is consistent with previous studies in other cell culture systems (Durán et al., 2012). The association of lactate and glutamine catabolism in the regulation of mTOR in these conditions of stressful vascular insufficiency is further substantiated by our results showing that mTOR upregulation can be suppressed by impairing lactate uptake with known inhibitors of MCT1, or by inhibiting enzymes involved in glutaminolysis, namely glutaminase 2 that converts glutamine to glutamate in the first step of its metabolism and an alanine aminotransferase that in turn catalyzes the production of alanine + α-ketoglutarate from (lactate-derived) pyruvate and glutamate. Other studies have reported that mTOR regulates glutamine flux and glutaminase levels (Csibi et al., 2014).

The observed upregulation/segregation of mTOR activity into the normoxic cell compartment induced by two potent AIs, and the resultant putative metabolic symbiosis, can be disrupted by concomitant inhibition of mTOR signaling with rapamycin. Remarkably, the initial impact of inhibiting mTOR in normoxic cancer cells is the necrotic death of the hypoxic cells, which lack elevated levels of mTOR signaling. An explanation for this intercellular crosstalk is offered by the observation that another glucose importer, GLUT2, is upregulated in normoxic peri-vascular cancer cells in response to rapamycin. The data suggest that the normoxic cancer cells proximal to the few remaining vessels consume the available blood-borne glucose when mTOR is inhibited, thereby starving distal hypoxic cancer cells, leading to their demise. Additionally, our NMR analysis of tumors suggests that mTOR inhibition blocks lactate catabolism via the anaplerotic pathway, and this abrogation of lactate consumption may lead to the intra-cellular and peri-cellular accumulation of toxic levels of lactate. The regulatory pathways involved in GLUT2 induction and metabolic reprogramming when the normoxic compartment is subjected to mTOR inhibition warrant future investigation.

Remarkably, as summarized in the introduction, multiple modes of adaptive or evasive resistance to therapies targeting tumor angiogenesis have been reported (Casanovas et al., 2005, Shojaei and Ferrara, 2008, Bergers and Hanahan, 2008, Ebos et al., 2009, Pàez-Ribes et al., 2009, Sennino et al., 2012) in experimental therapeutic trials performed using conventional transplant and genetically engineered de novo mouse models of cancer. Of these, only heighted invasiveness has been clearly implicated as an adaptive resistance mechanism in a human cancer, namely glioblastoma (Lu and Bergers, 2013; and references therein), although similar mechanisms can be envisioned to underlay the transitory clinical responses to AI therapy in virtually all tested forms of human cancer. Notably, the accompanying report by Jiménez-Valerio et al. (2016, this issue of *Cell Reports*) documents the induction of MCT1/MCT4 lactate transporters in a pattern consistent with metabolic symbiosis in renal cell carcinoma (RCC) patient-derived xenograph tumor (PDX) models treated with AI therapy. Moreover, analysis of biopsies of tumors from RCC patients treated with sunitinib produced histologic signatures of metabolic symbiosis that were exacerbated in relapsing/progressing (resistant) tumors from patients on anti-angiogenic therapy. Their data further show that this metabolic patterning is associated with TOR signaling, since its inhibition can evidently block this characteristic patterning. This study in human RCC, along with the accompanying article by Pisarsky et al. (2016, this issue of *Cell Reports*) in a mouse model of breast cancer and the results presented herein collectively and compellingly add metabolic symbiosis to the roster of adaptive/evasive resistance mechanisms to anti-angiogenic therapy.

In a broader context, the existence of metabolic symbiosis in tumors has been previously reported by Dewhirst, Sonveaux, Feron, and colleagues to arise spontaneously in certain transplant tumor models (Kennedy et al., 2013, Sonveaux et al., 2008, Semenza, 2008). They have described in a series of publications a metabolic symbiosis that conveyed decreased reliance on bioavailable glucose that limited the collateral damage from secreted lactate and consequent lactic acidosis, achieved by symbiotic cells importing and utilizing the lactate produced by glycolysis (Kennedy et al., 2013, Sonveaux et al., 2008, Semenza, 2008). The concept of metabolic symbiosis in neoplasia has been extended to interactions between cancer cells and cancer-associated fibroblasts endothelial cells, macrophages (Nakajima and Van Houten, 2013; and references therein), and also documented in pancreatic adenocarcinoma (Guillaumond et al., 2013). Its physiological basis lays in analogous symbiotic relationships operative in certain normal tissues, in particular brain and muscle (Draoui and Feron, 2011; and references therein). The concept can now be extended further, with the demonstrations that metabolic symbiosis can be induced in response to a serious environmental stress—angiogenesis inhibition, with consequent dropout and insufficiency of the tumor vasculature, and widespread hypoxia—in models of pancreatic neuroendocrine, breast, and renal cancer and in human RCC (this report, and the accompanying reports by Pisarsky et al. [2016] and Jiménez-Valerio et al. [2016]).

The question arises as to whether disruption of this mode of resistance might have value in extending the duration of efficacious responses by anti-angiogenic therapies. We show that metabolic symbiosis induced by sunitinib/axitinib can be disrupted by concomitant inhibition of mTOR signaling, resulting in significant reductions in TB and viability, but with only modest (albeit significant) extension in survival. The limited survival benefit suggests that “rapalogs” may not prove to be ideal drugs to disrupt this form of adaptive resistance; moreover, combinations of sunitinib/axitinib with the rapalog everolimus have been tested clinically, with limited benefit and significant toxicity (Molina et al., 2012). An alternative and potentially more attractive strategy may be to inhibit the MCT1 and/or MCT4 lactate transporters with highly selective drugs. Indeed, the preliminary studies reported herein and in the companion paper by Christofori et al. (2016), in which expression of MCT4 was suppressed genetically, encourages further preclinical and then potentially clinical evaluation, given that a number of MCT1 and MCT4 inhibitors are in pharmacological development.

An important further consideration is that tumors faced with vascular dropout and insufficiency evoked by potent AIs may activate multiple modes of adaptive/evasive resistance, in different regions of the tumor microenvironment. For example, in the RIP1Tag2 mouse model of PanNET, sunitinib is apparently inducing two distinctive modes of adaptive resistance, namely, symbiosis in hypoxic regions of regressing tumors, and, as reported previously, increased invasion and metastasis (Pàez-Ribes et al., 2009), in part by upregulating cMET signaling (Sennino et al., 2012). It is presently unclear whether these invading cancer cells also induce symbiosis upon reaching a size/location where diffusion of oxygen and glucose from co-opted normal vessels is insufficient, evoking hypoxia and, in turn, symbiosis. In fact, others have postulated that tumor acidity may promote local invasion (Gatenby and Gillies, 2004, Estrella et al., 2013). Interestingly, we observed that co-inhibiting angiogenesis and mTOR reduces the incidence of metastasis, but it is unclear whether this effect relates to disruption of symbiosis or some other physiological parameter. Future studies in preclinical and clinical trials may shed light on the interplay between adaptive resistance mechanisms and means to concomitantly disrupt them.

**Figure 7:**
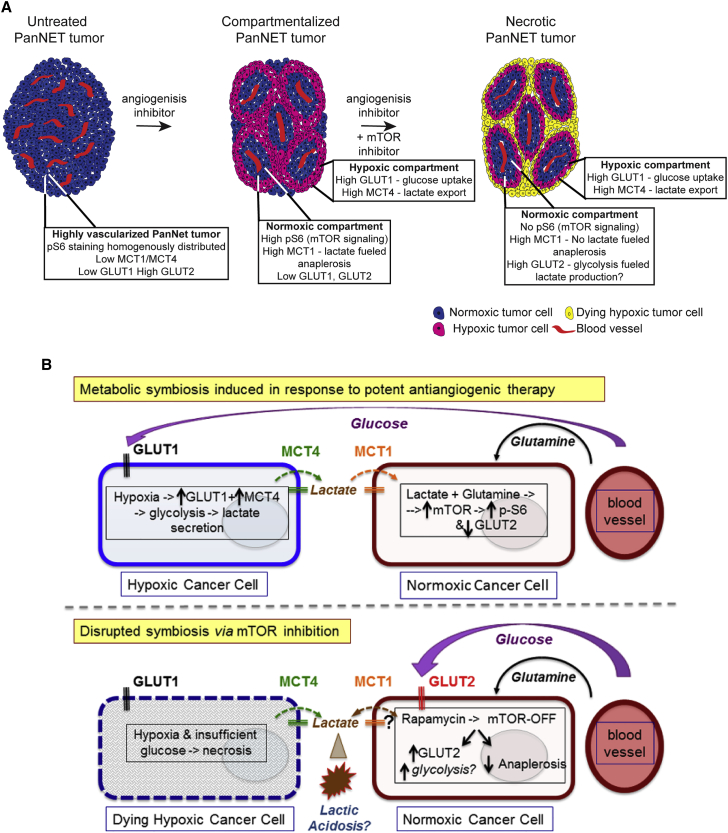
Schematic Conceptualizations of AI-Induced Metabolic Symbiosis (A) Untreated PanNET tumors are highly vascularized and express low levels of MCT1, MCT4, and GLUT1, but appreciable levels of GLUT2. Potent AIs targeting the VEGFR and PDGFR pathways elicit regression of the tumor vasculature with consequent regional hypoxia and tumor compartmentalization, marked by upregulation of MCT4 and GLUT1 in a hypoxic compartment and elevated levels of MCT1 and pS6 along with reduced levels of GLUT2 in a normoxic compartment. Combined inhibition of VEGFR/PDGFR and mTOR produces necrotic cell death in the hypoxic compartment, associated with GLUT2 upregulation and altered lactate metabolism in the normoxic compartment, followed by eventual necrosis. (B) Extensive vascular collapse resulting from AI treatment results in hypoxia that induces HIF1α, which in turn upregulates the glycolytic targets GLUT1 and MCT4 in the hypoxic cancer cells, leading to high levels of lactate secretion. Accumulating extracellular lactate induces expression of the MCT1 at the cell surface and lactate import into the normoxic compartment. In concert with serum-derived glutamine, lactate is catabolized in normoxic cancer cells with consequent induction of mTOR signaling to promote tumor metabolism. The data imply that normoxic cancer cells spare glucose for the hypoxic cells and fuel themselves by importing the lactate byproduct of glycolysis operative in the hypoxic cells in conjunction with glutamine. The bottom image suggests that metabolic symbiosis is disrupted by inhibition of mTOR through (indirect) upregulation of GLUT2 in the normoxic cells within the putative symbiotic clusters. Additionally, NMR studies indicate that mTOR inhibition disrupts the conversion of lactate-derived pyruvate/glutamate to alanine/α-ketoglutarate (anaplerosis) in dual-treated tumors, thereby disrupting the production of TCA cycle intermediates and potentially further enhancing toxic lactate accumulation.

An interesting question, not fully answered by the current studies, is how these metabolic clusters are induced to form, and in particular, why the peri-vascular normoxic cancer cells choose to take up and metabolize lactate and spare the limited glucose for their hypoxic brethren. A plausible explanation for the formation and disruption of metabolic symbiosis is illustrated schematically in Figure 7, and might transpire as follows: extensive vascular collapse elicited by the AIs results in hypoxia to trigger the HIF1α transcription factor; HIF1α induces GLUT1 and MCT4 and activates glycolysis in the hypoxic cancer cells, leading to high levels of lactate secretion; accumulating extracellular lactate induces expression of the MCT1 lactate transporter and, consequently, import of lactate into the normoxic cancer cells; in concert with serum-derived glutamine, lactate is catabolized in the normoxic cancer cells with consequent induction of mTOR signaling to promote tumor metabolism.

Notably, mTOR expression must be both cause and consequence, since its pharmacological inhibition results in the normoxic cancer cells upregulating a glucose transporter (GLUT2), and apparently switching to secrete rather than import lactate, resulting both in insufficient glucose for the hypoxic cells and likely toxic acidosis in the extracellular microenvironment. In addition, mTOR has been shown by others to be upregulated by (Durán et al., 2012) and also to control glutamine metabolism (Csibi et al., 2014). We extend these results by showing that its inhibition blocks the conversion of lactate-derived pyruvate/glutamate to alanine/α-ketoglutarate in R+S-treated tumors, disrupting the production of TCA cycle intermediates, potentially contributing to increased levels of (toxic and non-catabolized) lactate. While plausible, this rationale for the assembly and disruption of symbiosis will require future experimental validation.

In conclusion, the induction of metabolic symbiosis in response to vascular insufficiency elicited by potent AIs further illustrates the remarkable propensity of cancer cells to circumvent barriers that arise during tumorigenesis, tumor progression, and in response to treatment. This symbiotic adaptation evidently constitutes another mechanism that enables tumors to overcome the damaging effects of targeted therapies, by co-opting cellular processes intended for important homeostatic purposes that instead serve the evolving cancer.

## Experimental Procedures

### Therapeutic Agents

Sunitinib and axitinib were purchased from LC laboratories. Rapamune (rapamycin) was purchased from Galexis. Please see Supplemental Information for details on dosing regimens, formulations, etc.

### Mice

All mice used in this study were maintained in a pathogen-free barrier animal facility of the Swiss Federal Institute of Technology Lausanne (EFPL) in accord with Swiss regulations for the care and use of mice in experimental research.

### ^3–13^C-lactate and ^1^^–^^13^C-glucose in Tumor Cells

βTC3 cells were acclimated to “low” glucose (6.25 mM glucose + 4 mM Gln +10% FCS). They were subsequently plated at 7 × 10^6^ cells/10 cm tissue culture dish in low glucose culture media for 2 days, switched to 0% glucose/0% Gln + 10% FCS ON, then cultured in 20 mM ^3^^–^^13^C-lactate or 20 mM ^1^^–^^13^C-glucose + 4 mM Gln (no glucose) for 16 hr in hypoxic (1% O_2_/5% CO_2_) or normoxic (21% O_2_/5% CO_2_) conditions, and harvested for NMR. Plates were harvested individually on ice, rinsed twice with PBS, and collected in 0.9 M perchloric acid, which was subsequently titrated to approximately neutral pH with 0.9 M potassium hydroxide (KOH); extracts were lyophilized for NMR.

### ^3–13^C-lactate Uptake in Tumors

Mice were treated for 10 days with sunitinib or vehicle control and were infused with ^3^^–^^13^-C-lactate in PBS, by intraperitoneal (i.p.) injection, over a period of 90 min. Pentobarbital was administered, and the mice were perfused intravenously (i.v.) with a fluoresceinated lectin. Tumors were excised and immediately quick frozen in liquid nitrogen. For NMR, the frozen tumors were pulverized in liquid nitrogen, 0.9 M perchloric acid was added to the pulverized tumor tissue, rotated at 4°C for 2 hr, and titrated with 0.9 M KOH to a final of approximately pH 6–7.

### NMR Acquisition

NMR analysis was performed using a Bruker AVII 800 MHz spectrometer (18.8 T) equipped with a triple channel 5 mm cryoprobe (CPTCIz). 13C and 1H spectra were acquired using 30° angle pulses on 13C with decoupling on proton (zgpg). A repetition delay (d1) of 3 s and 2,048 scans (ns) was used. Spectra were processed with MestreNova software 10 using 5 Hz of line broadening. The chemical shift scale was calibrated with 3–13C lactate (21 ppm) as a reference, according to Shen et al. (1999) and Patel et al. (2005). To prepare samples for introduction into the NMR, 300 μl of pulverized cell solution was mixed with 100 μl of D2O (99.9% deuterated), shaken for 2 min, and directly poured into a 5 mm NMR tube.

### Statistical Analysis

Statistical analysis was performed using GraphPad Prism (GraphPad Software) software, and a log rank test was performed using software from the Broad Institute (http://bioinf.wehi.edu.au/software/russell/logrank/index.html). Due to the small sample size (as few as eight animals per treatment group) and the fact that not all the data are normally distributed, a non-parametric, suitable Mann-Whitney test was used (Figures 1A, 5B, 5C, S1A, S3A, S4B, and S4C; Tables S1 and S2), the log rank test was used for survival studies (Figure 5D), and the Student’s t test was used for Figures 4C, S5C, and S5D. For the incidence of metastasis during survival trials (Figure S3B), data were analyzed using R, where the event is “metastasis” and the horizontal axis is “age of sacrifice due to declining health status”, we tested whether the metastases are more frequent in one group versus the other (function “survdiff”).

## Author Contributions

E.A. and D.H. designed the study and wrote the manuscript. E.A. performed the experiments and analyzed the data. P.M. produced and analyzed the NMR data; C.M.W. analyzed/quantitated mTOR signaling in tumors; L.L. and M.-W.P. produced the RNA-seq data; and M.-W.P. contributed to all aspects of the preclinical studies and performed RT-qPCR experiments and ImageJ analysis. S.S. performed bioinformatics analysis.

## Accession Numbers

The accession number for the RNA-seq data reported in this paper is GEO: GSE80436.

## Supplemental Information

Document S1. Supplemental Experimental Procedures, Figures S1–S7, and Tables S1 and S2

Document S2. Article plus Supplemental Information
