# Supplementary material for: Metabolic Symbiosis Enables Adaptive Resistance to Anti-angiogenic Therapy that Is Dependent on mTOR Signaling
Source: Cell Rep. 2016 Apr 28;15(6):1144–60. doi: 10.1016/j.celrep.2016.04.029 (PMC4872464; doi:10.1016/j.celrep.2016.04.029)
Supplement: Document S1. Supplemental Experimental Procedures, Figures S1–S7, and Tables S1 and S2 [file mmc1.pdf]

Cell Reports, Volume *15*

Supplemental Information

Metabolic Symbiosis Enables Adaptive Resistance to Anti-angiogenic Therapy that Is Dependent on mTOR Signaling

Elizabeth Allen, Pascal Miéville, Carmen M. Warren, Sadegh Saghafinia, Leanne Li, Mei-Wen Peng, and Douglas Hanahan

Figure S1, related to Figure 2. Quantitation of vascularity in tumors treated from 13-15W. (A) Control untreated tumors showed characteristically high vascularity (top left), as did Mono-R (top right). Mono-S (top middle left), Mono-A (top middle right), and combination R+S (lower middle left) and R+A (lower middle right) show all have significantly reduced vascularity. Bottom panels depict DC101 (anti-VEGFR2 only, Mono-D) alone (bottom left) or in combination with rapamycin (R+D, bottom right). All mono- and combo therapy arms using AI produced significantly reduced vascularity versus controls or Mono-R, and all arms with sunitinib or axitinib produced significantly reduced vascularity versus those using DC101. 12-15 fields were scored from 2-3 mice/treatment group. Scale bars represent 100 μm. **(B) Quantitation of MCT1/MCT4 co-expression.** Ten treated tumors from 4 Mono-S or R+S mice were analyzed for co-expression of MCT1/MCT4 in strongly MCT4 positive tumor regions.

A

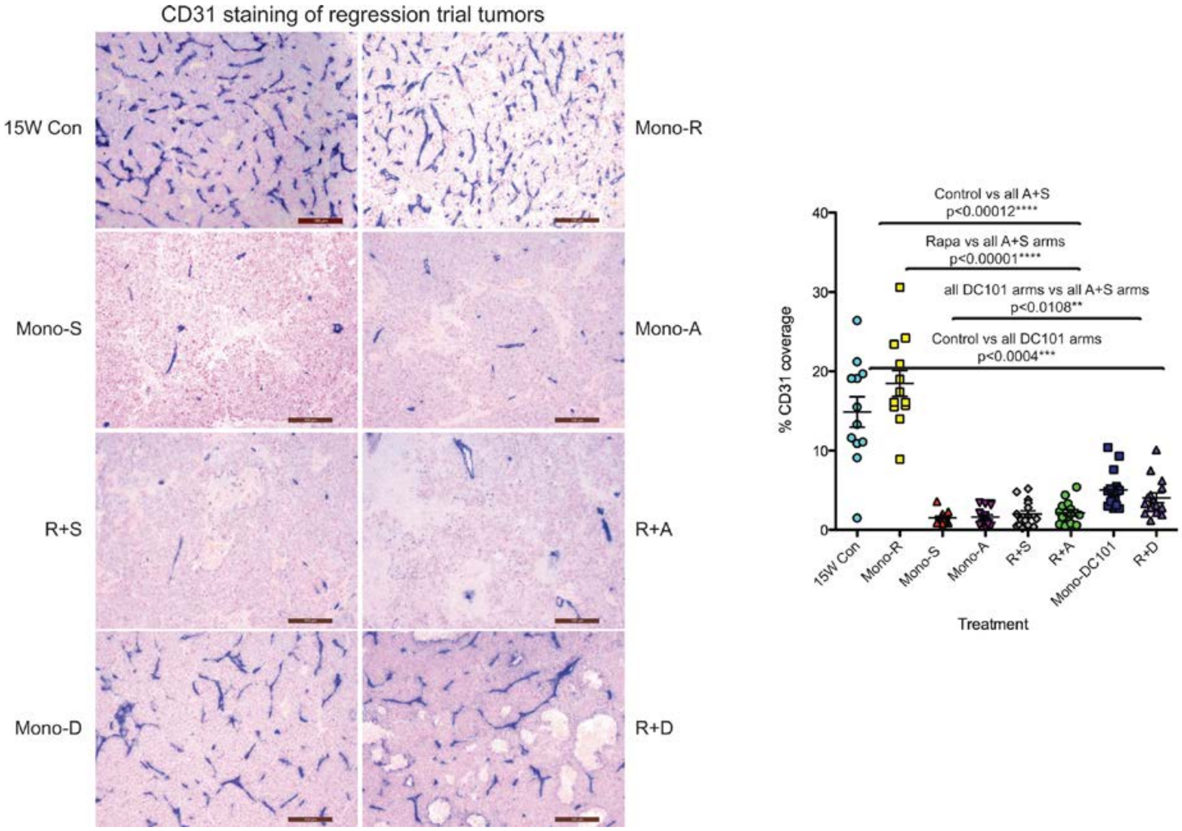

B

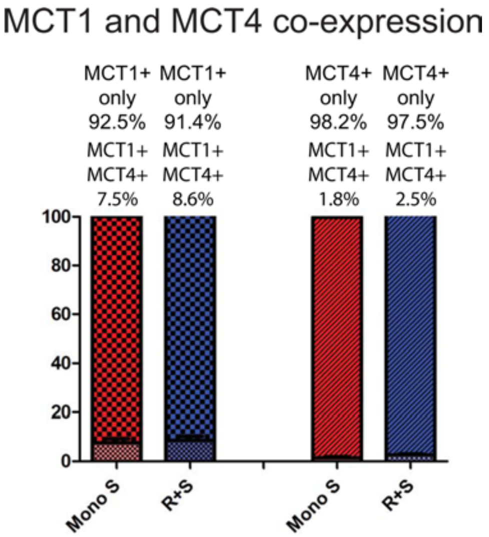

Figure S2, related to Figure 4. Schematic depicting lactate catabolism in treated tumors. (A) Proliferation of βTC3s in different carbon sources. βTC3s were cultured in differing combinations of glucose, glutamine, and lactate +/- rapamycin over 18H, and then assayed using a EdU incorporation assay to assess whether proliferation was effected. Little difference was found for all the arms that contained glutamine, but culture in glucose only +/- lactate produces a block in cell cycle progression. Data represents 3 replicates. **(B) Lactate reduction in CM in βTC3s** A scatter plot depicting the normalized reduction in lactate levels in CM for Gln only, Gln+Lactate, and Gln+Lactate + Rapa **(C) SiHa cells upregulate pS6/mTOR when cultured in lactate + glutamine, can be reversed by rapamycin or lactate uptake inhibition using CHC**. Lactate avid SiHa cells consumed lactate and upregulated pS6 when cultured in lactate and Gln, and this upregulation in Gln+lactate could be reversed rapamycin or the MCT1 inhibitor, 1mM CHC, which blocks lactate uptake. Below the blot are the values of the net reduction of lactate in conditioned media. **(D) Schematic depicting lactate catabolism in treated tumors.** 13C-lactate is converted to 13C-pyruvate, and enters the TCA cycle as acetyl-CoA to produce glutamate or aspartate, or replenishes TCA cycle intermediates (anaplerosis) by a transamination between pyruvate and glutamate that yields alanine and α-ketoglutarate. The 13C-lactate NMR results reveal that control, untreated tumors produce products of both pathways, while Mono-S favors the pathway that produces alanine, and R+S favors production of glutamate and aspartate. This schematic is adapted from Sybille Mazurek, https://commons.wikimedia.org/wiki/File:Glutaminolysisengl2.png.

A

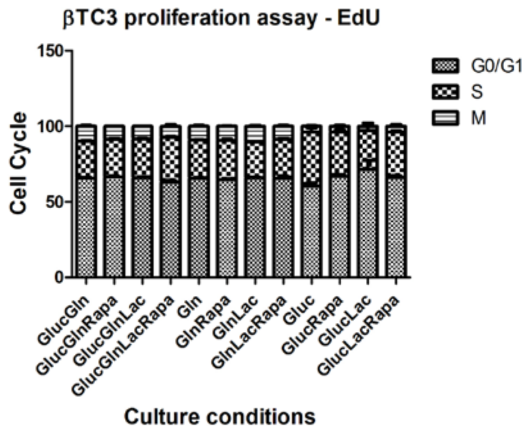

B

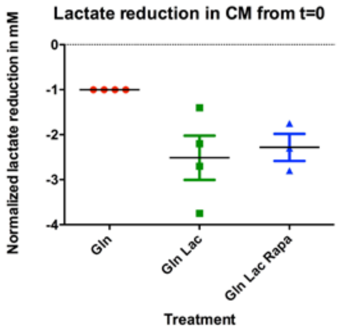

C

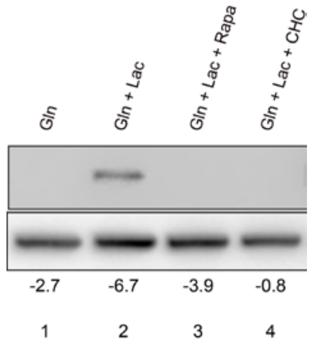

D

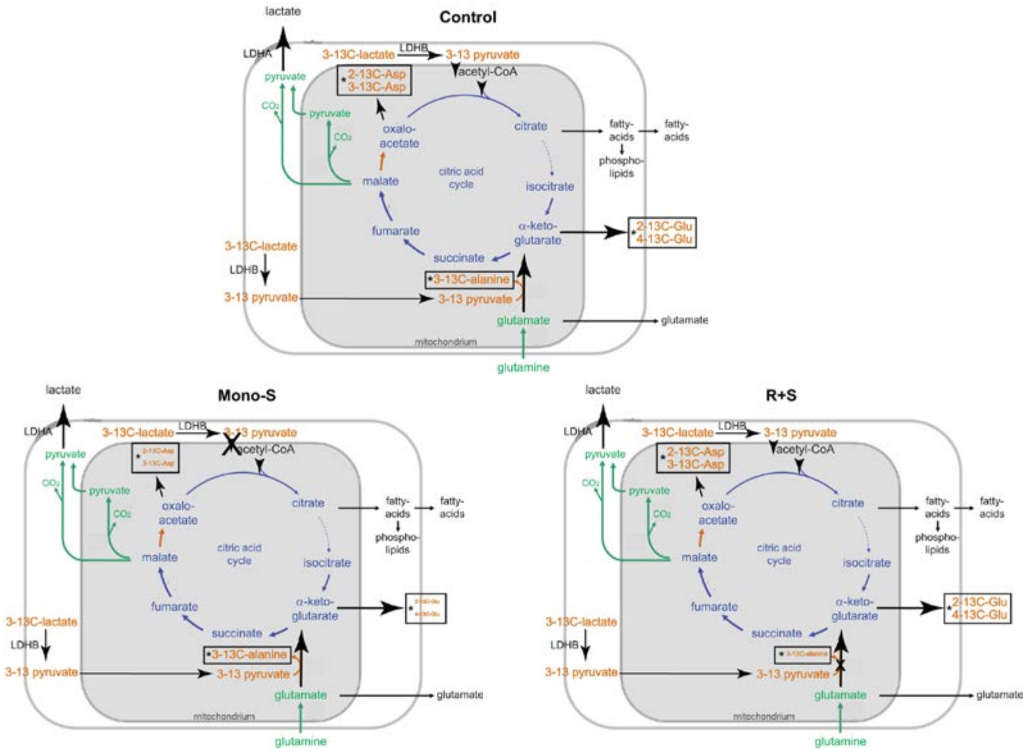

Figure S3, related to Figure 5. Tumor burden and metastasis in mice from open end survival trials.

(A) Tumor burden was assessed in end-stage mice that were exhibiting end stage signs (weight loss, lethargy, loss of appetite, low body temperature), with age indicated next to each point. Some R+S treated mice were healthy and survived until 30W, at which time they were sacrificed for evaluation, along with 2 healthy R+A animals at 28.6W. One 30W R+S Sim animal had very low tumor burden and no metastasis, whereas a second 30W R+S St animal had somewhat higher but still markedly reduced tumor burden, along with some micrometastasis. Due to low sample size, there are only statistical differences between Vehicle and Mono-S and Vehicle and R+S. Overall, tumor burden was higher in axitinib versus sunitinib therapies at end stage **(B)** Mice from Mono-S and Mono-A were combined as the "AI only" group and R+S and R+A groups were combined as "Rapa/AI". One end-stage rapamycin-treated mouse had macroscopic liver metastases, in contrast to vehicle and other treated arms, which only produced micro-metastases. The data was analyzed using the R package "survival" (function "survdiff"), and there was a very significant reduction in the incidence of micrometatasis in the Rapa/AI vs AI dosing arms.

A

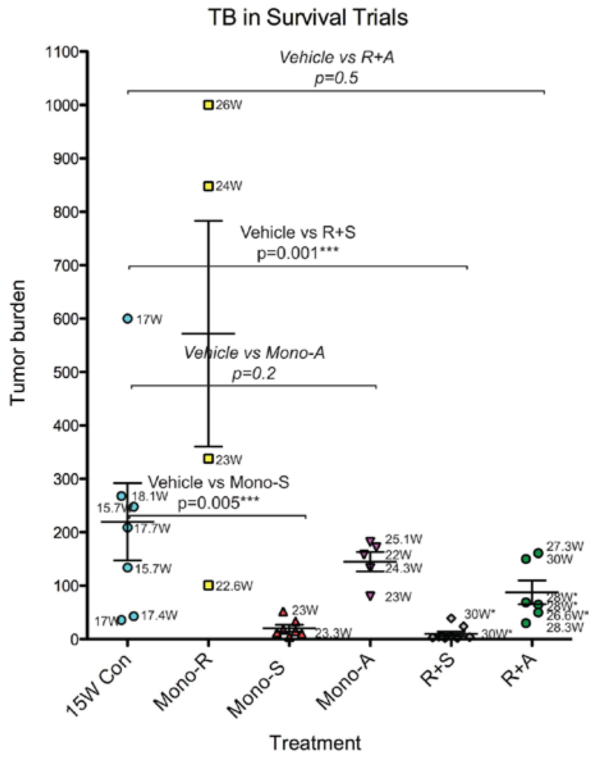

B

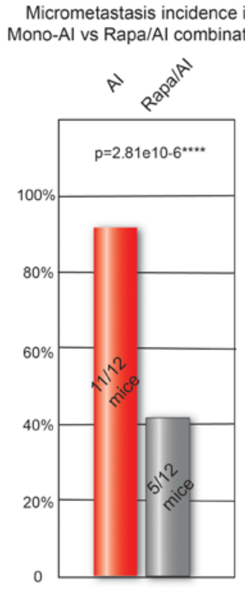

Figure S4, related to Figure 5. Combination therapy in treated tumors reduces proliferation versus other treatment arms.
(A) IHC analysis of 13-5W R+S tumor. HE staining of an R+S treated tumor produces markedly necrotic/fibrotic tumors with extensive immune infiltration and large swaths of fibrotic/necrotic tumor (upper panels, H&E), with vessels in regions devoid of TCs (CD31, arrows). In contrast to Mono-Su, (13-21W, depicted in Figure 1E) TC proliferation can sometimes be almost entirely absent in combination arms (Ki-67). Scale bar represents 700µm in the upper panel, 100µm in upper middle and bottom panels, and 200µm in lower middle panel. **(B) KI67 staining of 13-5W control or treated tumors.** Overall proliferation was comparatively quantitated for different treatment groups (right panels, Ki-67 staining), and representative images are depicted. There is a striking downregulation of proliferation in some combination treated tumors (R+S, 4th row panels), and an overall reduction in proliferation versus controls, in contrast to monotherapies which did not produce a significant difference. Scale bars represent 50 µm. **(C) Tunel staining of 13-5W control or treated tumors.** There is a highly significant increase in tunel staining in all treated arms versus control, but no significant difference in between treatment arms. Both tumor cell and endothelial cell tunel staining is scored, as in the rightmost Mono-S panel. Between 28-42 fields were scored from 2 different animals/treatment group. Scale bars represent 50 µm.

A

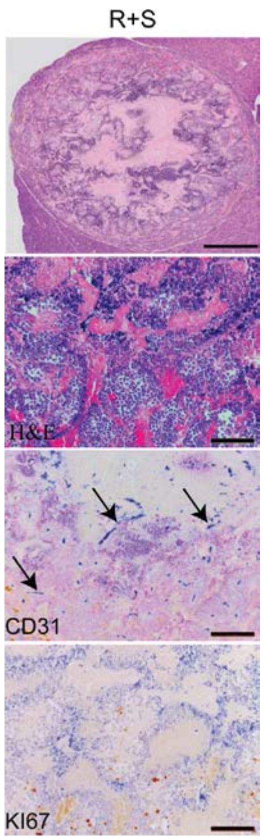

B

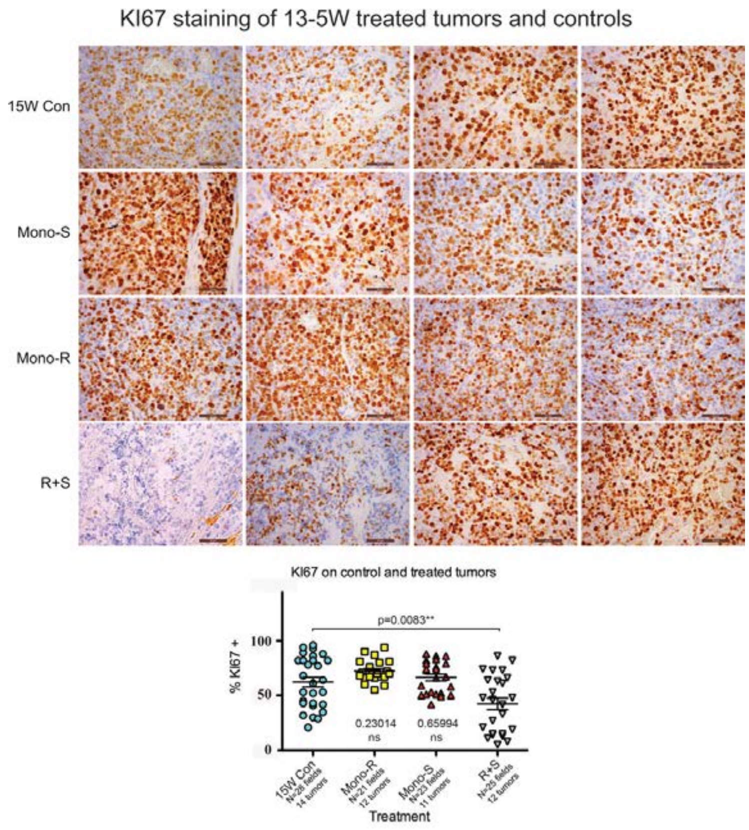

C

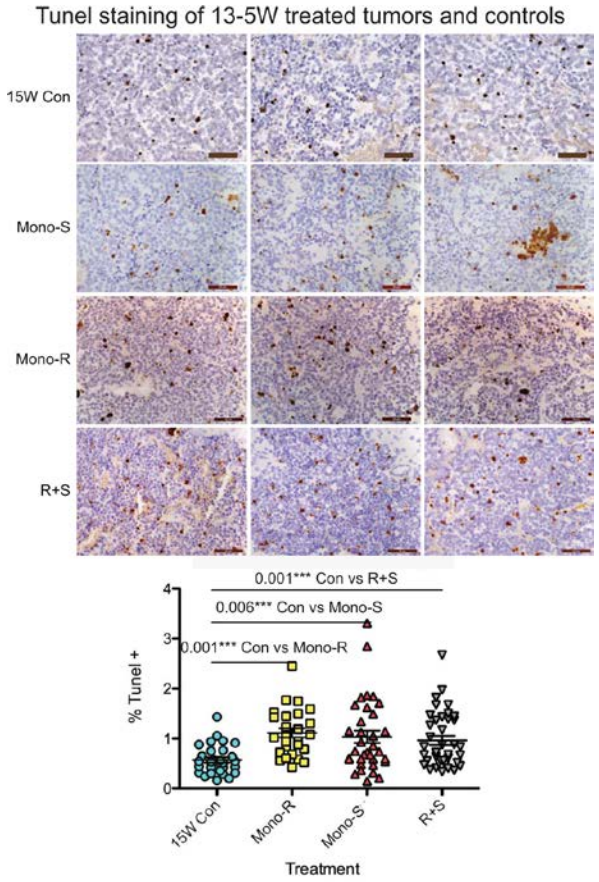

Figure S5, related to Figure 6. Heterogeneous GLUT1/GLUT2 expression in untreated Rip1Tag2 tumors. (A) In very large, hypoxic untreated Rip1Tag2 tumors, GLUT1 expression (blue) is upregulated in central hypoxic regions, while GLUT2 (brown) staining can be found in more normoxic regions in the tumor periphery. Notably, heterogenous staining of GLUT1/GLUT2 is detected in other control tumors (not shown). Scale bars represent 200µm in the top and 50µm in the bottom panels. **(B) Upregulation of GLUT2 in Mono-S vs R+S tumors, IHC.** Combination GLUT1 (blue) and GLUT2 (brown) IHC was performed under identical conditions using the Ventana automated staining system on 13-15W treated tumors from three different representative mice/group. In tumors with matched GLUT1 staining (blue), there is higher GLUT2 staining (brown) in regions flanking hypoxia in R+S treated tumors versus Mono-S**.** Scale bars represent 200µm**. (C) Upregulation of GLUT2 in Mono-S vs R+S tumors, western analysis.** The panel depicts western blot analysis of lysates from total treated tumors from Mono-S and R+S treated tumors, and GLUT2 levels are modestly upregulated. **(D) Upregulation of GLUT2 in glutamine+lactate+rapamyin versus glutamine+/-lactate, western analysis** The right panel depicts βTC3 cells cultured in Gln alone, Gln+Lac, or Gln+Lac+Rapa, and there was modest upregulation of GLUT2 receptor in the Gln+Lac+Rapa treated tumor cells. Statistical analysis was performed using a one-tailed Student's T-test.

A

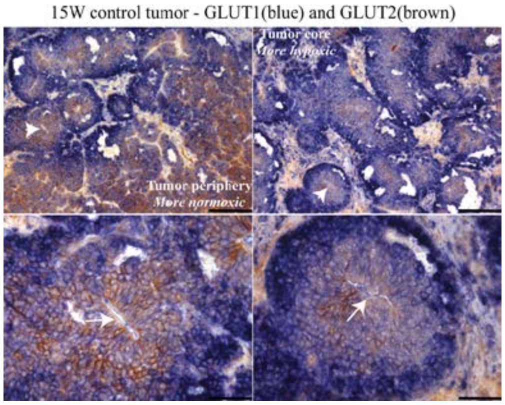

B

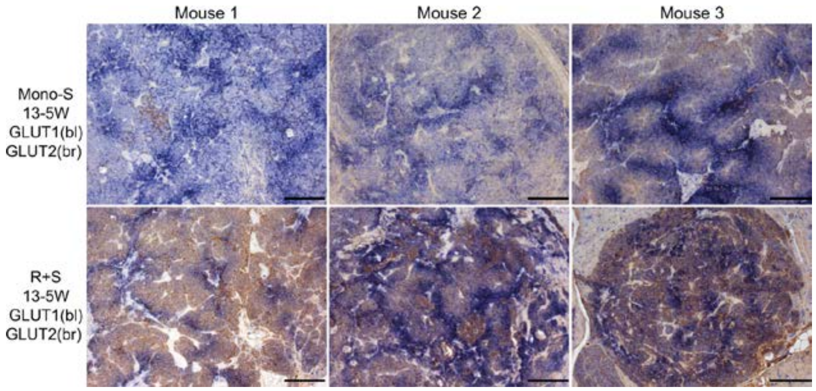

C

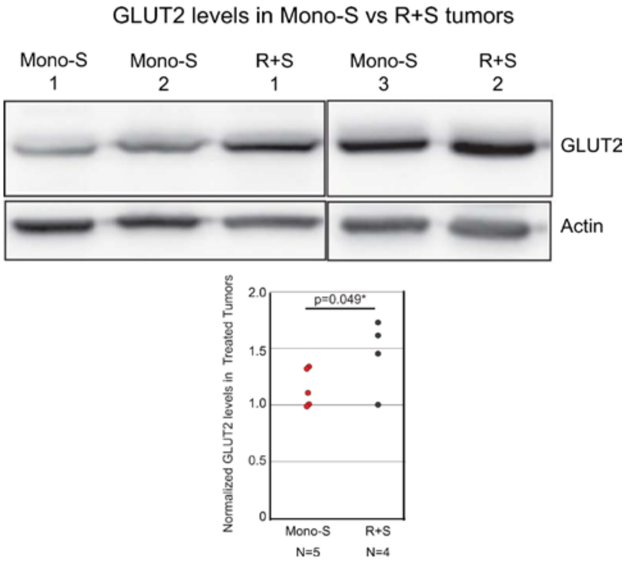

D

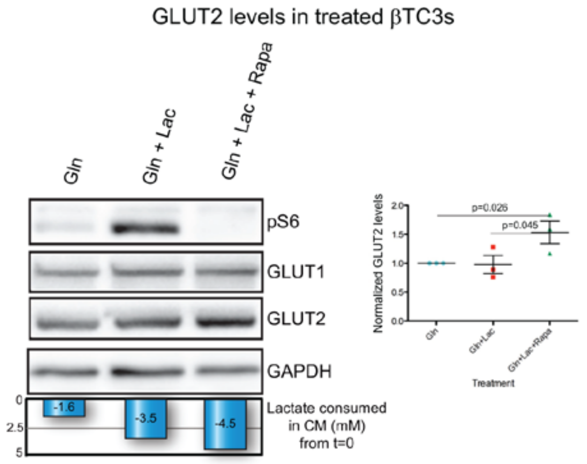

Figure S6, related to Figure 6. Ultrastructural analysis of necrotic regions of R+S combination treated tumors.
(A) Ultrastructural analysis by EM of an osmium stained R+S tumor with extensive necrosis/fibrosis (upper right, black arrowheads show tumor border). Transmission electron microscopic (TEM) images were taken and overlapped to image the vessel (middle) with associated viable perivascular TCs, extending to the dying TCs at the fibrotic border. Bottom panels show images of the granular and fibrotic tumor regions (bottom left) and the viable TCs that border these regions (bottom right). In the right panel, black arrows indicate relatively healthy mitochondria clustered around the nucleus, while in both panels dashed arrows indicate swollen and degenerating mitochondria, and arrowheads indicate insulin granules. In the right panel, purple arrows show the borders of a degenerating cell membrane from a cell that contains relatively healthy mitochondria clustered around the nucleus, with cellular contents spilling into the surrounding dying, fibrotic tissue. The highly necrotic tissue has markedly distended mitochondria in comparison to that in the adjacent cell. See also Figure S4A and panel B. Scale bars are 2mm in the middle and lower panels. **(**B) Panels depict additional images from tissue within the R+S treated islet tumors. The left panel shows cells abundant with insulin granules (arrow heads), and granulated cytoplasm with swollen mitochondria (dashed arrows) that contain flocculent densities. Right-hand panel depicts another region where this degeneration extends to a blood capillary where an endothelial cell (EC) appears normal.

A

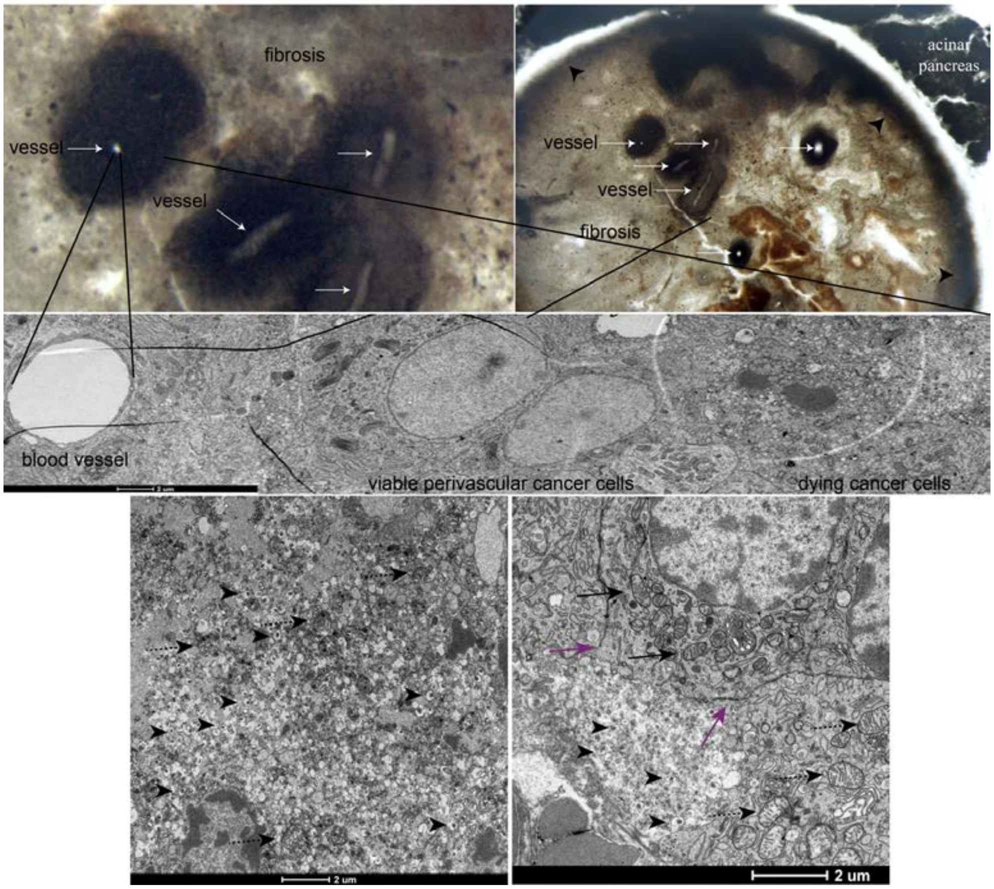

B

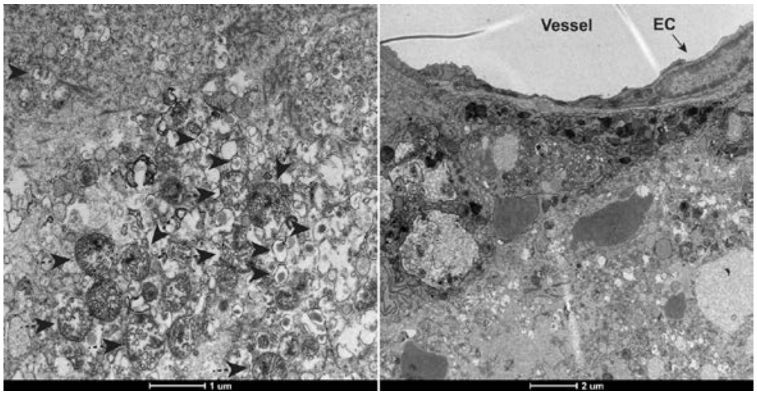

Figure S7, related to Figure 6. A comprehensive summary of the antibody staining of MCT4/MCT1, pimonidazole with pS6, GLUT2, and GLUT1 in Control, Mono-S and R+S tumors. MCT4/MCT1 (left, top) reactivity is strongly upregulated in Mono-S and R+S treated tumors versus Controls, with very strong MCT1 staining in the R+S panels. Pimo/pS6 (left, bottom). Control, non-hypoxic (Pimo-) tumors have homogenous pS6 staining, while Mono-S tumors have compartmentalized pS6 staining in the normoxic regions, and no pS6 reactivity in R+S treated tumors. GLUT2/Pimo (top, right). GLUT2 expression is highest in normoxic Control tumors, with lower expression in R+S treated tumors, and the lowest expression in Mono-S tumors. GLUT1/Pimo (bottom, right) GLUT 1 expression is lowest in non-hypoxic Control tumors, with much higher expression in Mono-S and R+S tumors. In R+S tumors, the pimo+ hypoxic regions are smaller in the R+S treated tumors than in Mono-S, although there is residual GLUT1+ staining, supporting the notion that the hypoxic regions are dying first in the R+S tumors. The scale bar represents 50μm.

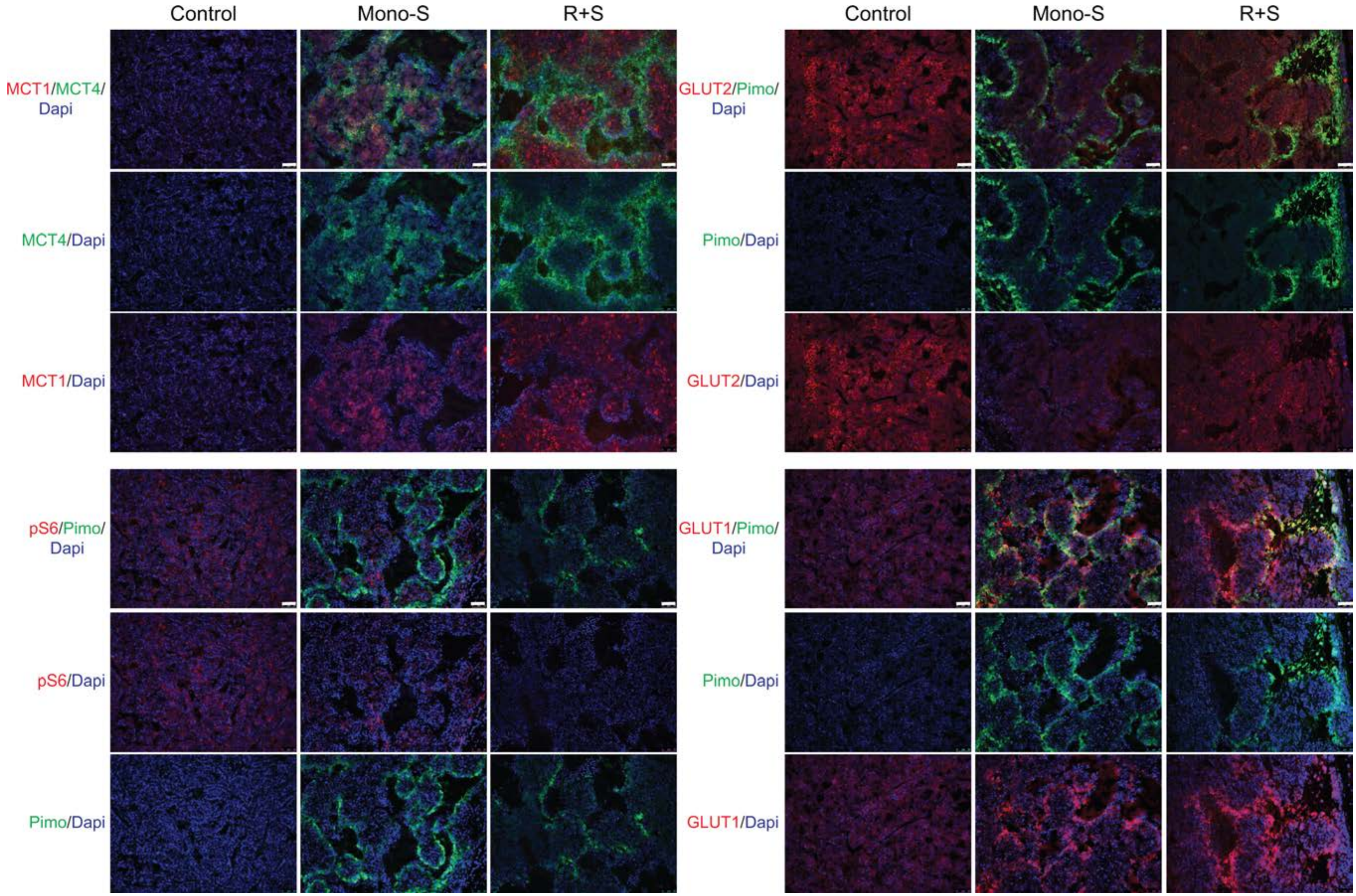

Table S1.

a. RNAseq Results, related to Figure 2.

Treatment	Sample #	Gene	Fold Change	Adj pVal
Control	4	*Slc2a1*/GLUT1	1.00	
Mono-S	4		2.20	0.004
R+S	5		1.11	*0.09*
Control	4	*Slc2a2*/GLUT2	1.00	
Mono-S	4		0.55	*0.07*
R+S	5		0.63	*0.17*
Control	4	*Gpt2*/	1.00	
Mono-S	4	Alanine aminotransferase 2	1.38	0.008
R+S	5		0.35	*0.03*
Control	4	*Gls2*/	1.00	
Mono-S	4	Glutaminase 2	1.43	0.028
R+S	5		0.88	*0.83*
Control	4	*Ldha*/	1.00	
Mono-S	4	Lactate dehydrogenase A	7.13	0.004
R+S	5		0.95	*0.72*
Control	4	*Slc16a3*/MCT4	1.00	
Mono-S	4		14.19	0.004
R+S	5		3.34	0.002
Control	4	*Slc16a1*/MCT1	1.00	
Mono-S	4		10.78	0.03
R+S	5		1.50	*0.28*

Gene expression data was derived from RNAseq analysis from 4-5 tumors from each treatment group, and adjusted pValue is shown.

b. RT-qPCR Results, related to Figure 2.

Treatment	Sample #	Gene	Fold Change	pValue
Control	5	*Slc2a1*/GLUT1	1.00	-
Mono-S	5		4.54	0
R+S	6		3.46	0
Control	5	*Slc2a2*/GLUT2	1.00	-
Mono-S	5		0.39	0
R+S	6		0.50	0.0003
Control	5	*Gpt2*/	1.00	-
Mono-S	5	Alanine aminotransferase 2	1.52	0.028
R+S	6		0.89	*0.171*
Control	5	*Gls2*/	1.00	-
Mono-S	5	Glutaminase 2	1.72	0.007
R+S	6		1.88	0.028
Control	5	*Ldha**	1.00	-
Mono-S	5	Lactate Dehydrogenase A	2.73	0.0009
R+S	6		0.96	*0.689*
Control	5	*Slc16a3*/MCT4	1.00	-
Mono-S	5		5.11	0
R+S	6		1.39	0.033
Control	5	*Slc16a1*/MCT1	1.00	-
Mono-S	5		2.63	0.183
R+S	6		1.12	*0.833*

Gene expression data was derived from Q-RT-PCR analysis using 5-6 tumors from each treatment group with 1 primer pair/gene, and both Rpl19 and Rpl13 normalization controls, (or *Rpl19 and GAPDH). Samples were analyzed in triplicate for each gene, and data was analyzed using a 2-tailed Mann-Whitney test.

Table S2. RT-qPCR Results from βTC3 cells under normoxia/hypoxia, related to Figure 4.

Treatment	n	Gene	Expression Ratio	pValue
			Hyp/Norm	
Gluc/Gln	6	*Slc2a1*/GLUT1	5.85	0.0051
Gluc/Gln/Lac			5.48	0.0051
Gln			3.79	0.0051
Gln/Lac			2.66	0.0051
Gluc			5.76	0.0051
Gluc/Lac			5.30	0.0051
			Norm/Hyp	
Gluc/Gln	6	*Slc2a2*/GLUT2	2.77	ns
Gluc/Gln/Lac			4.06	0.0083
Gln			6.15	0.0080
Gln/Lac			6.28	0.0080
Gluc			4.35	*0.0658*
Gluc/Lac			4.84	0.0203
			Hyp/Norm	
Gluc/Gln	6	*Slc16a3*/MCT4	7.06	0.0051
Gluc/Gln/Lac			6.53	0.0051
Gln			5.46	0.0051
Gln/Lac			3.11	0.0051
Gluc			8.31	0.0051
Gluc/Lac			7.11	0.0051
			Norm/Hyp	
Gluc/Gln	6	*Slc16a1*/MCT1	0.89	ns
Gluc/Gln/Lac			0.78	ns
Gln			1.27	ns
Gln/Lac			1.12	ns
Gluc			1.12	ns
Gluc/Lac			1.34	ns
			Hyp/Norm	
Gluc/Gln	6	*Ldha/*Lactate dehydrogenase A	10.62	0.0051
Gluc/Gln/Lac			10.03	0.0051
Gln			15.92	0.0051
Gln/Lac			5.61	0.0051
Gluc			27.14	0.0051
Gluc/Lac			17.76	0.0051
			Norm/Hyp	
Gluc/Gln	6	*Ldhb/* Lactate dehydrogenase B	1.35	0.0131
Gluc/Gln/Lac			1.12	ns
Gln			1.89	0.0051
Gln/Lac			1.81	0.0051
Gluc			1.42	ns
Gluc/Lac			1.33	0.0460
			Norm/Hyp	
Gluc/Gln	6	*Gls2*/Glutaminase 2	0.65	ns
Gluc/Gln/Lac			0.85	ns
Gln			0.81	ns
Gln/Lac			1.65	ns
Gluc			1.28	ns
Gluc/Lac			1.48	ns
			Norm/Hyp	
Gluc/Gln	6	*Gpt2*/Alanine aminotransferase 2	1.71	0.0203
Gluc/Gln/Lac			1.73	ns
Gln			2.35	0.0080
Gln/Lac			2.39	0.0203
Gluc			0.97	ns
Gluc/Lac			0.92	ns

Six combinations of media with distinct carbon sources were cultured in normoxia or hypoxia and used for RT-qPCR analysis using Rpl19 as a control, and this was repeated once and the results combined; data was for each carbon source was normalized, and then the ratio of expression in hypoxia versus normoxia was calculated. Statistics were generated using a 2-tail Mann-Whitney. ns= not significant

Supplemental Experimental Procedures.

Mice and trial design

The generation and characterization of the single transgenic RT2 (RIP1-Tag2) mice has been previously described (Hanahan 1985). Briefly, RT2 mice undergo multifocal stepwise tumorigenesis, producing hyper- and dysplastic islets, a subset of which subsequently undergo an angiogenic switch, leading in turn to formation of highly angiogenic PNET starting around 10 week; mice die at 15-16 week with a burden of 5-15 independent large, red, hemorrhagic PNET. Trial designs utilized in this study (intervention, fixed and open-ended regression/survival) are depicted in Figure 4A.

Therapeutic trials

RT2 mice were treated starting at 11, 13 or 14W with the following therapeutic regimens on a schedule of 6D per week: 1) Sunitinib (LC laboratories, Woburn MA, USA) was prepared once weekly at 10 mg/ml for the free base, and administered by oral gavage at 40 mg/kg in a vehicle formulation of 0.5% Carboxymethylcellulose sodium (USP, 0.5% w/v), 1.8% NaCl (USP, 1,8% w/v), 0.4% Tween 80 (NF, 0.4% w/v) and 0.9% benzyl alcohol (NF, 0.9% w/v); 2) axitinib (LC laboratories, Woburn MA, USA and Medchemexpress Co, Ltd.) was prepared at 10 mg/ml once weekly in a vehicle formulation of in 0.5% carboxymethylcellulose and administered by oral gavage twice daily as monotherapy at 30 and 40 mg/kg or once daily at 40 mg/kg in combination with rapamycin; 3) Rapamume (Galexis-AG, Ecublen, Switzerland) was administered by oral gavage at a dose of 5 mg/kg/day; 4) anti-VEGFR2 blocking antibody (DC101) was obtained in bulk by affinity purification from the supernatant of a hybridoma culture (DC101) available from American Type Culture Collection (ATCC) or was purchased from the UCSF Monoclonal Antibody Core, 513 Parnassus Ave., San Francisco, CA, USA, and administered at 1 mg/animal, twice weekly through intra-peritoneal injection as previously described; control mice were gavaged once daily with the sunitinib vehicle formulation for a period of time equivalent to the corresponding therapeutic regimens.

Histopathological analyses, hypoxia and lectin perfusion; western blot analyses

Pancreas, spleen and liver were obtained following PFA perfusion, cryoprotected in 30% sucrose, OCT embedded and frozen; non-perfused tissue was fixed in buffered formalin and embedded in paraffin, and antigen retrieval was performed with citrate. For immunohistochemistry or IF, sections were blocked in 4% NDS, 4% NMS, and 0.1% bovine serum albumin prior to addition of the primary antibody. The following antibodies were used for specific tissue immunostaining: Rabbit anti-GLUT1 (07-1401) and rabbit anti-GLUT2 (07-1402) were from Millipore, goat anti-MCT1 (T19, sc-14917), rabbit anti-MCT4 (H90, sc-50329), rat monoclonal anti-mouse CD31 (clone MEC13.3, 1:50, BD-PharMingen); rat anti-mouse CD31 (SZ31, DIA310, Dianova) rabbit polyclonal serum anti-large T antigen (1:10,000, preparation from the Hanahan laboratory); and rat anti-MECA32 antibody (1:200, 550563, BD-Pharmingen), phospho-S6 Ribosomal Protein Antibody Ser 235/236 (2211L), rabbit anti-GLS2 (AV43562-100ug, Sigma-Aldrich). FITC-, Rhodamine-, and HRP conjugated secondary antibody were obtained from Jackson ImmunoResearch (West Grove PA) and used according to the manufacturer's instruction. The following secondary antibodies were used at a dilution of 1:200: Alexa Fluor 488 goat anti-rat IgG and Alexa Fluor 546 goat anti-rabbit IgG. For specific staining of the nuclei samples were mounted with Vectashield with DAPI (Vector labs). To analyze functional vasculature, FITC-labeled tomato lectin (Vector labs, 0.1 mg in 0.1 ml) was injected intravenously and allowed to circulate for 5-10 min. Mice were anesthetized, heart perfused with neutral buffered formalin and PBS, and tissue was excised and OCT embedded for frozen sectioning. Detection of hypoxia was performed using pimonidazol (pimo) (60mg/kg), which was injected i.p. to the mice an hour before they were anesthetized, heart perfused with formalin, and tissue excised and OCT embedded for frozen sectioning. For the pimonidazol immunodetection, Hypoxyprobe-FITC conjugated antibody was used following manufacturer's instructions (Natural Pharmacia International Inc., Burlington MA).

Western blot analysis utilized β-actin (Abcam, ab8226, 1:2000) Monoclonal Anti-ß-Actin antibody (A5441, Sigma-Aldrich) and monoclonal anti-α-tubulin antibodies (T5168-.2ML). Rabbit anti-GLUT1 (07-1401) and rabbit anti-GLUT2 (07-1402) were from Millipore. Survivin, (2808S), Phospho-S6 Ribosomal Protein Antibody Ser 235/236 (2211L), and rabbit anti-GAPDH (2118S) were from Cell Signaling Technology, Danvers MA, USA. For western blots in Figure S4C, quantitation was performed using the LI-COR system and software (LI-COR Biosciences, Germany), and statistics were performed using a one-tail T-Test.

IHC using the Ventana automated system

CD31 protocol. Immunohistochemical detection of endothelial cells (rat α-CD31, clone SZ1, Dianova, diluted 1:100) was performed using the fully automated Ventana Discovery XT (Roche Diagnostics, Rotkreuz, Switzerland). All steps were performed on the machine with Ventana solutions. Briefly, dewaxed and rehydrated paraffin sections were pretreated with heat using mild condition (20 minutes) CC1 solution. The primary antibodies was incubated 1 hour at 37°C. After incubation with a donkey α-rat biotin (Jackson

ImmunoResearch Laboratories), chromogenic revelation was performed with BlueMap kit (Roche Diagnostics, Rotkreuz, Switzerland) for 60min. Tissue was counterstained with Nuclear Fast Red (Carl Roth) for 30 seconds.

Protocol double IHC. Double immunohistochemical detection of rabbit anti GLUT1 (07-1401, Millipore, diluted 1:200) and rabbit anti-GLUT2 (07-1402, Millipore, diluted 1:200) was performed using the fully automated Ventana Discovery XT (Roche Diagnostics, Rotkreuz, Switzerland). All steps were performed on the machine with Ventana solutions. Briefly, dewaxed and rehydrated paraffin sections were pretreated with heat using standard conditions (36 minutes) CC2 solution. The GLUT2 antibody was incubated 1 hour at 37°C. After incubation with the ready to use anti Rabbit OmniMap (Roche Diagnostics, Rotkreuz, Switzerland) chromogenic revelation was performed with DabMap kit (Roche Diagnostics, Rotkreuz, Switzerland). Antibodies were denatured for 8 minutes at 95°C before incubating Glut1 antibody for 1 hour at 37°C. Donkey α-rabbit biotin (Jackson ImmunoResearch Laboratories) was incubated before the chromogenic revelation was performed with BlueMap kit (Roche Diagnostics, Rotkreuz, Switzerland) for 60 minutes.

Tumor measurements

RT2 mice were euthanized and tumor volume (in mm^3) was measured from freshly excised pancreata by using the formula [volume = 0.52 x $(width)^2$ x (length)], approximately the volume of a sphere.

Glucose and lactate measurements

Serum glucose levels were monitored using the Bayer Breeze2 glucose meter. Lactate levels in conditioned media of cultured cancer cells were measured using the Lactate Pro 2 kit (Axon labs).

α-Ketoglutarate assay

One million cells ßTC3 cells were plated 6.25mM glucose + 4mM Gln +FCS in 1 well of a 6-well dish, grown for 2D, shifted ON to media with 0% glucose/0% Gln + FCS, and plated in DMEM +/- glucose, +/- Gln, and +/- lactate. Cells were harvested on ice in RIPA buffer + protease inhibitors, sonicated, and insoluble material removed by centrifugation. Extracts were quantitated for protein level using the Qubit system (Lifetechnolgies), and analysed for α-ketoglutarate levels using the α-KG assay kit (Abcam). The assay was repeated 3 times to produce 6 values each for Gln vs Gln + Lac, and relative measures are displayed and analyzed using a 2 tailed T-Test.

Tumor vascularity

Control or treated mice (13-15W) were analyzed for tumor vascularity by immunostaining with CD31 (blue) using the Ventana automated staining system (see above). For microvessel area quantification, either entire tumors or tumor regions up to 250 μm X 300 μm were randomly chosen from treated/untreated tumors, and ratio of CD31-stained area to total area was calculated in each spot using image analysis software, ImageJ (National Institute of Health, Washington DC, USA). The microvessel area (MVA) of the region was expressed as the average ratio of these calculations, and the total number of tumors/regions analyzed and mice utilized are shown beneath each treatment group in Figure S3, and data was analyzed using the Mann-Whitney test.

RNA-seq experiments

RNA quality was assessed with the Advanced Analytical Fragment Analyzer and samples with RNA quality number (RQN) above 7 were selected for library preparation and sequencing. RNA-seq libraries were generated with the Illumina TruSeq Stranded protocol, with polyA selection. The libraries were sequenced on an Illumina HiSeq2500 machine, generating a minimum of 40 million reads per sample. After sequencing, a further quality control was performed by analyzing the mapped read distribution along the gene length. Samples with a strong 5'-3' bias in read coverage variation were removed from the dataset, as this bias is indicative of RNA degradation (Khrameeva and Gelfand, 2012).

RNA-seq data processing

The RNA-seq reads were aligned on the mouse genome (assembly version mm10/GRCm38, downloaded from the Ensembl (Flicek et al., 2014) database release 74) using TopHat (Kim et al., 2013) release 2.0.13 and Bowtie2 (Langmead and Salzberg, 2012) release 2.1.0. The alignments were processed to extract uniquely mapping reads, which were used for all further analyses.

Enrichment analysis

To conduct the Gene Set Enrichment Analysis on the expression data, we proceeded with two separate data preparation and consecutive analytical methods as the following;

1) All the gene expression data from various treatment condition were collected and applied to the online GSEA software (Subramanian , et. al). Hallmark Gene Set collection were chosen from MSigSB as the reference for gene sets enrichment analysis.

2) To gain insight on common processes, pathways, and underlying biological themes that are produced from different treatments, gene expression values were sorted based on the fold change differences between control (no treatment) and treatment groups; the top 2500 genes with a pValue <0.05 which had the highest increase in their expression were selected and examined their overlap to different gene sets (adopted from Hallmark Gene Set).

Transmission electron microscopy of pancreatic tissue

Rip1Tag2 mice (14.5-15W of age) were treated as indicated for 7D, perfused with via the heart with 200 ml of 2.5 % glutaraldehyde and 2.0 % paraformaldehyde in 0.1M phosphate buffer (pH 7.4). After 2 hours the tissue was removed, and after embedding in 5% agarose, vibratome sliced at a thickness of 80 microns. Slices containing appropriate regions of interest were then washed thoroughly with cacodylate buffer (0.1M, pH 7.4), postfixed for 40 minutes in 1.0 % osmium tetroxide with 1.5% potassium ferrocyanide, and then another 40 minutes in 1.0% osmium tetroxide alone. The slices were finally stained for 30 minutes in 1% uranyl acetate in water, before being dehydrated through increasing concentrations of alcohol and embedded in Durcupan ACM (Fluka, Switzerland) resin. The sections were then placed between glass microscope slides coated with mold releasing agent (Glorex, Switzerland) and left to harden for 24 hours in a 65°C oven. Regions of interest were cut away from the rest of the tissue sections, glued to blank resin blocks with cyanocrylate glue, and thin (50 nm thick) sections cut with a diamond knife. These were collected onto pioloform support films on single slot copper grids, contrasted with lead citrate and uranyl acetate, and images taken with a transmission electron microscope at 80 kV (Tecnai Spirit, FEI Company with Eagle CCD camera).

EdU incorporation assay

The EdU incorporation assay from Invitrogen, Carlsbad CA, USA, was utilized (cat# A10044).

References

Hanahan D. (1985). Heritable formation of pancreatic beta-cell tumours in transgenic mice expressing recombinant insulin/simian virus 40 oncogenes. Nature *315*, 115-22.

Langmead B and Salzberg SL. (2012). Fast gapped-read alignment with Bowtie 2. Nat Methods. 9(4):357-9.

Kim D, Pertea G, Trapnell C, Pimentel H, Kelley R, Salzberg SL. (2013). TopHat2: accurate alignment of transcriptomes in the presence of insertions, deletions and gene fusions. Genome Biol. 14(4), R36.

Subramanian A, Tamayo P, Mootha VK, Mukherjee S, Ebert BL, Gillette MA, Paulovich A, Pomeroy SL, Golub TR, Lander ES, Mesirov JP. (2005). Gene set enrichment analysis: a knowledge-based approach for interpreting genome-wide expression profiles. Proc Natl Acad Sci U S A. 102(43):15545-50.

Inventory of Supplemental Information

Figure S1, related to Figure 2. Quantitation of vascularity and MCT1/MCT4 co-expression vs exclusivity in tumors.

Figure S2, related to Figure 4. Proliferation of βTC3s in different carbon sources, lactate uptake from CM, SiHa cells upregulated pS6 via lactate/glutamine catabolisme, schematic depicting lactate catabolism in treated tumors.

Figure S3, related to Figure 5. Tumor burden and metastasis in mice from open end survival trials.

Figure S4, related to Figure 5. Effects of Combination therapy in treated tumors on morphology, proliferation, and apoptosis versus other treatment arms.

Figure S5, related to Figure 6. GLUT1/GLUT2 expression in untreated and treated Rip1Tag2 tumors.

Figure S6, related to Figure 6. Ultrastructural analysis of necrotic regions of R+S combination treated tumors.

Figure S7, related to Figure 6. A comprehensive summary of the antibody staining of MCT4/MCT1, pimonidazole with pS6, GLUT2, and GLUT1 in Control, Mono-S and R+S tumors.

Table S1. RNAseq Results, related to Figure 2.

Table S2. RT-qPCR Results from bTC3 cells under normoxia/hypoxia, related to Figure 4.

Supplemental Experimental Procedures and References.
